# Workplace adjustments equivalent to reasonable accommodations and workplace maladjustment in a general working population, with a focus on high autism spectrum disorder (ASD) and attention deficit/hyperactivity disorder (ADHD) traits

**DOI:** 10.1265/ehpm.26-00080

**Published:** 2026-09-17

**Authors:** Tomoko Suzuki, Michiko Nakazato, Toshiyuki Ohtani, Shunya Ikeda

**Affiliations:** 1 Department of Public Health, School of Medicine, International University of Health and Welfare, 4-3 Kozunomori, Narita, Chiba 286-8686, Japan; 2 Department of Psychiatry, School of Medicine, International University of Health and Welfare, 4-3 Kozunomori, Narita, Chiba 286-8686, Japan; 3 Safety and Health Organization, Chiba University, 1-33 Yayoi-cho, Inage-ku, Chiba-shi, Chiba 263-8522, Japan

**Keywords:** Attention deficit/hyperactivity disorder (ADHD), Autism spectrum disorder (ASD), Reasonable accommodation, Workplace maladjustment, Worker

## Abstract

**Background:**

The potential effectiveness of reasonable accommodations for workers with autism spectrum disorder (ASD) and attention deficit/hyperactivity disorder (ADHD) has been reported, but quantitative evidence remains limited. It is also unclear whether ASD- or ADHD-equivalent workplace adjustments benefit workers with lower ASD or ADHD trait levels in the general population. This study aimed to quantitatively examine whether individuals with high or non-high levels of ASD or ADHD traits experience greater workplace maladjustment in environments without ASD- or ADHD-equivalent adjustments compared with those where such adjustments are provided.

**Methods:**

Participants were 1,000 randomly selected workers from across Japan who completed a self-administered online survey conducted as a cross-sectional study. Workplace adjustments equivalent to reasonable accommodations were identified from 14 examples drawn from two case collections published by the Ministry of Health, Labour and Welfare. ASD traits, ADHD traits, and workplace maladjustment were assessed using the Autism-Spectrum Quotient, the Adult ADHD Self-Report Scale, and the Social Adaptation Self-evaluation Scale, respectively. Logistic regression analysis was performed to estimate odds ratios and 95% confidence intervals for workplace maladjustment.

**Results:**

Among workers with high ASD or ADHD trait scores, the odds of workplace maladjustment were higher when ASD- or ADHD-equivalent adjustments were not provided than when such adjustments were available; a comparable but less pronounced association was also observed among those with non-high trait scores.

**Conclusions:**

These quantitative findings suggest that workplace adjustments equivalent to reasonable accommodations are associated with a lower risk of workplace maladjustment and improved workplace adaptation among employees with high ASD or ADHD trait levels, and may also be beneficial for those with milder traits.

**Supplementary information:**

The online version contains supplementary material available at https://doi.org/10.1265/ehpm.26-00080.

## Background

Autism spectrum disorder (ASD) is a neurodevelopmental disorder characterized by impairments in social communication, social interaction, and the presence of restricted and repetitive behaviors [1]. Attention-deficit/hyperactivity disorder (ADHD) is a chronic neuropsychiatric disorder with core symptoms of inattention, hyperactivity, and impulsivity [2]. These neurodevelopmental conditions often lead to difficulties in various aspects of daily and occupational functioning. Therefore, individuals with high levels of ASD or ADHD traits are generally considered to be socially disadvantaged. For example, high ASD or ADHD traits are more prevalent among workers with low socioeconomic status [3–6]. Additionally, individuals with high levels of ASD or ADHD traits are more likely to have depressive symptoms/depression [7–9]. Indeed, individuals with ASD often experience low and unstable employment [10]. Moreover, ADHD has been associated with underachievement at work [11] and frequent job changes [12].

However, in workplace settings, employees with ASD can thrive when provided with appropriate supports and an enabling environment, contributing a wide range of skills, distinctive perspectives, and high levels of productivity to their organizations [13]. Similarly, employees with ADHD can perform well and thrive when provided with appropriate workplace support [14]. These findings highlight the importance of workplace conditions that actively support neurodivergent employees, particularly through the implementation of reasonable accommodation. Reasonable accommodation is defined by the UN Convention on the Rights of Persons with Disabilities (CRPD) as “necessary and appropriate modification and adjustments not imposing a disproportionate or undue burden, where needed in a particular case, to ensure to persons with disabilities the enjoyment or exercise on an equal basis with others of all human rights and fundamental freedoms” [15]. While the CRPD provides a broad legal definition of reasonable accommodation, empirical research has proposed more operational definitions. In research on autistic employees, Petty et al. defined reasonable accommodations as measures that positively impact their well-being and job performance without adversely affecting non-autistic employees or the organization, and that are low cost and easy to implement [16].

Although several reports have documented examples of reasonable accommodation, including case collections published by the Ministry of Health, Labour and Welfare [17], most existing evidence is derived from case studies or qualitative research, with limited quantitative evaluation. To address this evidence gap, we quantitatively examined workplace adaptation levels and the perceived need for adjustments equivalent to reasonable accommodations for ASD or ADHD in the general population.

Most prior research on reasonable accommodation has focused on individuals with diagnosed ASD or ADHD or those with clearly elevated levels of these traits, and it remains unclear whether benefits of ASD- or ADHD-equivalent workplace adjustments extend to workers with lower levels of ASD or ADHD traits in the general working population. This perspective is consistent with the concept of universal design in the workplace, which emphasizes creating work environments that accommodate diversity and promote inclusion, thereby benefiting not only individuals with pronounced neurodevelopmental traits but also the broader workforce. Universal design, as defined by the United Nations Development Programme, refers to “the design of products, environments, programmes, and services to be usable by all people, to the greatest extent possible, without the need for adaptation or specialized design” [18].

This study aimed to quantitatively examine whether workers with high or non-high ASD or ADHD trait levels differ in perceived workplace maladjustment according to the presence or absence of workplace adjustments equivalent to reasonable accommodations. We also examined whether they differ in perceived need for such adjustments.

## Methods

### Participants

Participants comprised Japanese workers who completed a self-administered, Internet-based questionnaire survey conducted as a cross-sectional study in March 2025. The participant flowchart is presented in Supplementary Fig. 1. The sample was drawn from a panel of individuals registered with a Japanese online survey company (Rakuten Insight Inc., Tokyo, Japan), which comprises 279,808 registered members across all Japanese prefectures. A total of 6,624 registered members were randomly selected and invited to participate in the study. Screening was conducted on the basis of the following eligibility criteria: aged 20–59 years and currently employed. Of these, 2,345 members responded to the screening, and 1,305 met the eligibility criteria and were granted access to the self-administered questionnaire. The survey system was designed to automatically submit responses upon completion, ensuring no missing data as all items had to be answered to finalize the survey. When the survey closed, 1,000 respondents were randomly selected by the survey company, and their responses were provided for analysis. This group of 1,000 participants constituted the final analytic sample. To control for potential bias related to sex and age, the sample was balanced by both attributes: the male-to-female ratio was 1:1, and equal numbers of participants were included in each of the following age groups: 20–29, 30–39, 40–49, and 50–59 years. This equal allocation was adopted to minimize the confounding effects of age and sex on the outcomes. Proportional allocation based on the actual working population would have resulted in underrepresentation of certain age–sex strata, reducing statistical precision and comparability across groups. Workers aged 60 years and older were excluded because the normal retirement age in Japan is 60 or 65 years.

### Sociodemographic characteristics

Sociodemographic characteristics were obtained via a self-administered questionnaire. These comprised age, sex, marital status (married or unmarried), socioeconomic status (annual household income, educational attainment, job type, and occupational position), and health-related behaviors (smoking status, drinking frequency, and leisure-time physical activity). Annual household was divided income into five groups (<3, 3–5, 5–8, 8–10, and ≥10 million yen; 150 yen ≈ 1 USD); educational attainment into three groups (high school or less, postsecondary non-university education, and university degree or higher); smoking status into three groups (current, former, or never smoker); drinking frequency into three groups (rarely, sometimes, or almost every day); and leisure-time physical activity into two groups (“exercise,” defined as light activity ≥1 time/week to intense activity ≥3 times/week, vs. “no exercise”). Job type was categorized into six groups: company employee, professional (e.g., physician, lawyer, accountant), government employee, self-employed, contract/temporary worker, and part-time worker. Among these, employment status was classified into three groups: regular employment (company employee, professional, and government employee), non-regular employment (contract/temporary worker and part-time worker), and self-employed. Occupational position was categorized into four groups: executive/business owner/president, director/executive officer/department head, section manager/assistant manager, and staff level/non-managerial position.

### Measures

Workplace adjustments equivalent to reasonable accommodations for neurodevelopmental disorders were assessed using 14 items derived from two case collections published by the Ministry of Health, Labour and Welfare: one collection on reasonable accommodations for persons with disabilities, including those with neurodevelopmental disorders [17], and another collection focusing specifically on neurodevelopmental disorders [19]. The items were compiled by reviewing multiple publicly available case examples and then consolidating them into 14 representative questions using an iterative process. The final set was judged to capture the main types of workplace adjustments relevant to neurodevelopmental disorders, including communication support, workload adjustment, sensory/environmental accommodation, scheduling flexibility, and supervisory consultation. These items were selected because they represent practical workplace adjustments commonly reported in official Japanese case examples and collectively cover major domains of accommodation relevant to workers with neurodevelopmental disorders. The 14 items are listed in Supplementary Table 1. For this study, workplace adjustments equivalent to reasonable accommodations were defined as adjustments that are equivalent to those required under the reasonable-accommodations framework, regardless of whether an employee has a formally diagnosed neurodevelopmental disorder. Workplace adjustments equivalent to reasonable accommodations were assessed using two sets of items: (1) whether each type of adjustment was implemented and (2) whether the worker perceived it as necessary. For implementation, respondents selected one of four options: ‘implemented,’ ‘partially implemented,’ ‘rarely implemented,’ or ‘not implemented.’ For analysis, the first two categories were combined as ‘implemented’ and the latter two as ‘not implemented.’ For perceived necessity, respondents selected one of five options: ‘strongly necessary,’ ‘necessary,’ ‘depends,’ ‘rarely necessary,’ or ‘not necessary.’ For analysis, the categories were grouped into three levels: ‘strongly necessary,’ ‘necessary,’ and the remaining three categories.

The Autism-Spectrum Quotient (AQ) is a self-report questionnaire that assesses ASD traits in adults of normal intelligence [20]. It consists of 50 items rated on a four-point Likert scale (“definitely agree,” “slightly agree,” “slightly disagree,” and “definitely disagree”) across five subscales (10 items each): social skills, attention switching, attention to detail, communication, and imagination. For half the items, responses of “definitely agree” or “slightly agree” are scored as 1, whereas “slightly disagree” or “definitely disagree” are scored as 0; the remaining items are reverse-scored to minimize response bias. Total scores range from 0 to 50, with higher scores indicating greater ASD trait levels. A psychometric evaluation confirmed good test-retest reliability and internal consistency of the AQ. In the original study, test–retest reliability showed a strong correlation (r = 0.70). This study used the Japanese version translated by Wakabayashi et al. [21], which demonstrated similarly high reliability (r = 0.87) and has established validity and reliability. Cutoff scores suggesting likely ASD are ≥32 (original English version) and ≥33 (Japanese version). The validity of the cutoff scores has been supported by quantitative indices reported in previous studies. In the original English version, a cutoff of ≥32 correctly identified 79.3% of individuals with Asperger syndrome and high-functioning autism (AS/HFA; corresponding to ASD in current diagnostic criteria) while misclassifying only 2% of controls, indicating high sensitivity and specificity [20]. In the Japanese version, a cutoff of ≥33 identified 87.8% of individuals with AS/HFA (now classified under ASD) and misclassified only 3% of controls, demonstrating similarly high discriminative validity [21]. Participants were thus classified into two groups: the high ASD trait group (AQ ≥ 33) and the non-high group (AQ < 33).

The adult ADHD Self-Report Scale (ASRS) [22] was used to assess participants’ ADHD traits. This study employed the validated Japanese version of the ASRS [23]. The ASRS is an 18-item self-report questionnaire that screens for the 18 DSM-IV [24] symptoms of adult ADHD. The six-item ASRS Screener, a validated short form, was specifically used in this study. Participants rated how often each symptom occurred over the past 6 months. Total scores range from 0 (no symptoms) to 6 (severe symptoms), with scores ≥4 indicating suspected ADHD. The validity of this cutoff has been supported by previous studies. The 6-item ASRS Screener has been shown to have high specificity (99.5%) and acceptable sensitivity (68.7%) for identifying adults with ADHD in validation studies, indicating strong discriminative performance [22]. Accordingly, a score of ≥4 was used to define suspected ADHD in the present study. Participants were therefore classified into two groups: the high ADHD trait group (ASRS ≥ 4) and the non-high group (ASRS < 4).

The Social Adaptation Self-evaluation Scale (SASS) [25] was used to assess participants’ workplace maladjustment. The SASS is a scale developed to evaluate patients’ social motivation and behavior in depression and is widely used to assess social functioning. It consists of 21 items covering different domains of life, including work, leisure, family and non-family relationships, intellectual interests, satisfaction with social roles, and self-perceived ability to manage and control one’s environment. Each item is scored from 0 to 3, with higher scores indicating better social functioning; the total score ranges from 0 to 60. This study used the validated Japanese version of the SASS [26]. A cut-off score of ≤25 has been suggested as an indication of social impairment [26]. The validity of this cutoff has been supported by the Japanese validation study, which identified a cutoff of 25/26 as the optimal threshold and reported a sensitivity of 0.93 and a specificity of 0.51 [26]. In this study, workplace maladjustment was defined as having a SASS score of 25 or below.

### Sample size calculation

The sample size was calculated based on the odds ratio (OR) for workplace maladjustment associated with developmental disorders. Because the prevalence of workplace maladjustment in the general working population has not been well established in previous literature, we used the prevalence of depressive symptoms as a proxy, given the strong negative correlation between depressive symptoms and workplace maladjustment (ρ = −0.683, p < 0.001) [26].

We referenced a previous study [27] to determine the calculation parameters. The parameters were as follows:

• The prevalence of ASD-equivalent individuals (high ASD trait levels, AQ ≥ 33) was assumed to be 5% of the working population.
• The prevalence of depressive symptoms among individuals with non-high ASD trait levels (AQ < 33) was approximately 11%.
• The OR for depressive symptoms among individuals with high ASD trait levels ranged from 3 to 16, depending on the type of job stress.

To ensure a conservative estimate, we adopted an OR of 3 (the minimum effect size reported), thereby securing sufficient statistical power even under stricter assumptions. With α = 0.05 (two-sided) and 80% power, the required sample size was calculated to be 910 participants using the OR test for two-bin proportions.

### Statistical analyses

Descriptive statistics were performed to summarize participant characteristics and the implementation and perceived necessity of workplace adjustments equivalent to reasonable accommodations. Logistic regression analysis was conducted to estimate the odds ratios (ORs) and 95% confidence intervals (CIs) for workplace maladjustment. Two logistic regression models were constructed, each using a four-category grouping defined by trait level (high vs. non-high) and the presence or absence of reasonable accommodation–equivalent adjustments. In both models, workers with non-high trait scores who received reasonable accommodation–equivalent adjustments served as the reference group. One model examined ASD traits, and the other examined ADHD traits. Univariate analyses were first performed, followed by multivariable logistic regression adjusted for age, sex, marital status, annual household income, educational attainment, job type, occupational position, smoking status, drinking frequency, and leisure-time physical activity. Subsequently, we conducted stratified analyses by employment status and by sex. For the stratified analyses by employment status, we excluded self-employed individuals and performed analyses separately for regular and non-regular employees. Using a chi-square test, we compared the perceived necessity of reasonable accommodation–equivalent workplace adjustments between workers with high versus non-high ASD traits and between those with high versus non-high ADHD traits. Regarding perceived necessity, responses were categorized into three groups—strongly necessary, necessary, and other—considering the possibility that some respondents might select “necessary,” even when adjustments were not strictly required, if they perceived potential benefits. “Other” comprised the response options: “case-dependent,” “not strongly necessary,” and “not necessary.” Two-tailed p-values < 0.05 were considered statistically significant. All analyses were conducted using SPSS Statistics for Windows, version 24.0 (IBM Corp., Armonk, NY, USA).

### Ethics statement

Ethical approval was obtained from the International University of Health and Welfare Ethics Committee (No. 24-TA-143). The study was conducted in accordance with the principles of the 1964 Declaration of Helsinki and its later amendments. Completion of the anonymous online questionnaire was considered as indicating informed consent, and the requirement for written informed consent was waived by the International University of Health and Welfare Ethics Committee.

## Results

Participant demographics are shown in Table 1. Table 2 shows implementation proportions and perceived necessity of reasonable accommodation–equivalent workplace adjustments. The proportions of implementation for several types of workplace adjustments equivalent to reasonable accommodations ranged from approximately 40% to 70%. Similarly, the proportions of workers who strongly perceived the necessity of these adjustments ranged from approximately 7% to 20%. Among all workplace adjustments, consideration for health or flexible work hours was the most commonly implemented (n = 682, 68.2%) and was also most strongly perceived as necessary (n = 193, 19.3%).

**Table 1:** Participants’ characteristics.

Sex	Male	500 (50.0)
Age group (years)	20–29	250 (25.0)
	30–39	250 (25.0)
	40–49	250 (25.0)
	50–59	250 (25.0)
Marital status	Married	465 (46.5)
Annual household income (million yen)	<3	146 (14.6)
	3–5	259 (25.9)
	5–8	306 (30.6)
	8–10	127 (12.7)
	≥10	162 (16.2)
Educational Attainment	High school or less	235 (23.5)
	Postsecondary non-university education	203 (20.3)
	University degree or higher	562 (56.2)
Job type	Company employee	606 (60.6)
	Professional (e.g., physician, lawyer, accountant)	35 (3.5)
	Government employee	76 (7.6)
	Self-employed	63 (6.3)
	Contract / Temporary worker	59 (5.9)
	Part-time worker	161 (16.1)
Employment status	Regular employment	717 (71.7)
	Non-regular employment	220 (22.0)
	Self-employed	63 (6.3)
Occupational position	Executive / Business owner / President	30 (3.0)
	Director / Executive officer / Department head	52 (5.2)
	Section manager / Assistant manager	121 (12.1)
	Staff level / Non-managerial position	797 (79.7)
Smoking status	Never smoker	690 (69.0)
	Former smoker	149 (14.9)
	Current smoker	161 (16.1)
Drinking frequency	Rarely	456 (45.6)
	Sometimes	408 (40.8)
	Almost every day	136 (13.6)
Leisure-time physical activity	No exercise	581 (58.1)
	Exercise	419 (41.9)
ASD trait scores	High (AQ ≥ 33)	78 (7.8)
ADHD trait scores	High (ASRS ≥ 4)	98 (9.8)
Work Maladjustment	(SASS ≤ 25)	272 (27.2)

n (%); ASD: autism spectrum disorder; ADHD: attention-deficit/hyperactivity disorder; AQ: Autism-Spectrum Quotient; ASRS: adult ADHD Self-Report Scale; SASS: Social Adaptation Self-evaluation Scale.

**Table 2:** Implementation and perceived necessity of reasonable accommodation–equivalent workplace adjustments.

**No.**	**Workplace adjustments**	**Implementation**	**Perceived necessity**		

			**Strongly necessary**	**Necessary**	**Other**
1	Adjustment of Break Times	582 (58.2)	93 (9.3)	314 (31.4)	593 (59.3)
2	Quiet Rest Area	515 (51.5)	135 (13.5)	372 (37.2)	493 (49.3)
3	Consideration for Health / Flexible Work Hours	682 (68.2)	193 (19.3)	379 (37.9)	428 (42.8)
4	Workload Sharing / Reducing Burden	567 (56.7)	132 (13.2)	389 (38.9)	479 (47.9)
5	Dedicated Space for Focused Work	398 (39.8)	101 (10.1)	304 (30.4)	595 (59.5)
6	Clear and Specific Work Instructions	604 (60.4)	144 (14.4)	378 (37.8)	478 (47.8)
7	Multiple Modes of Instruction Delivery	571 (57.1)	105 (10.5)	377 (37.7)	518 (51.8)
8	Advance Notice of Schedule Changes	530 (53.0)	139 (13.9)	399 (39.9)	462 (46.2)
9	Consideration for Task Focus	491 (49.1)	95 (9.5)	344 (34.4)	561 (56.1)
10	Regular Meetings with Supervisors	518 (51.8)	77 (7.7)	322 (32.2)	601 (60.1)
11	Task Assignment Considering Individual Strengths	515 (51.5)	106 (10.6)	373 (37.3)	521 (52.1)
12	Consideration for Sensory Stimuli	523 (52.3)	114 (11.4)	345 (34.5)	541 (54.1)
13	Active Workplace Communication	623 (62.3)	123 (12.3)	372 (37.2)	505 (50.5)
14	Training on Diversity and Disability Awareness	419 (41.9)	69 (6.9)	284 (28.4)	647 (64.7)

n (%); “Other” includes responses of “case-dependent,” “not strongly necessary,” and “not necessary.”

Table 3 presents the associations of ASD traits or ADHD traits and reasonable accommodation–equivalent workplace adjustments with workplace maladjustment. The reference group was workers with non-high ASD trait scores and the presence of adjustments for the ASD analyses, and workers with non-high ADHD trait scores and the presence of adjustments for the ADHD analyses. The results of the analysis after adjustment for possible confounding factors were as follows. Workers with non-high ASD or ADHD trait scores and the absence of reasonable accommodation–equivalent adjustments showed significantly higher odds of workplace maladjustment (ORs: 1.82–3.71) compared with the respective reference groups, and all types of reasonable accommodation–equivalent adjustments were statistically significant. Among workers with high ASD trait scores, the presence of reasonable accommodation–equivalent adjustments was associated with ORs of 3.94–7.30, all of which were statistically significant, whereas the absence of such adjustments was associated with markedly higher odds (ORs: 10.51–22.88, all significant). Among workers with high ADHD trait scores, the presence of reasonable accommodation–equivalent adjustments was associated with ORs of 1.84–4.70, all statistically significant except for one adjustment (Quiet rest area), while the absence of such adjustments was associated with ORs of 6.71–13.24, all of which were statistically significant.

**Table 3:** Associations of ASD traits, ADHD traits, and reasonable accommodation–equivalent workplaces with workplace maladjustment.

**(A) ASD traits**									

**No.**	**Workplace adjustments**		**ASD** **traits**	**No. of** **subjects**	**No. of** **cases**	**Univariate**		**Multivariable**	
	
	**Type**	**Presence**				**OR (95% CI)**	**p-value**	**OR (95% CI)**	**p-value**
1	Adjustment of Break Times	Yes	Non-high	544	88	1.00		1.00	
		No	Non-high	378	132	2.78 (2.04–3.80)	<0.001	2.82 (2.02–3.93)	<0.001
		Yes	High	38	22	7.13 (3.60–14.11)	<0.001	6.48 (3.11–13.47)	<0.001
		No	High	40	30	15.55 (7.33–32.95)	<0.001	13.88 (6.3–30.55)	<0.001
2	Quiet Rest Area	Yes	Non-high	483	82	1.00		1.00	
		No	Non-high	439	138	2.24 (1.64–3.06)	<0.001	2.32 (1.67–3.23)	<0.001
		Yes	High	32	17	5.54 (2.66–11.55)	<0.001	4.69 (2.14–10.27)	<0.001
		No	High	46	35	15.56 (7.59–31.9)	<0.001	14.74 (6.93–31.35)	<0.001
3	Consideration for Health / Flexible Work Hours	Yes	Non-high	630	113	1.00		1.00	
		No	Non-high	292	107	2.65 (1.94–3.62)	<0.001	2.64 (1.89–3.68)	<0.001
		Yes	High	52	30	6.24 (3.47–11.22)	<0.001	5.50 (2.94–10.26)	<0.001
		No	High	26	22	25.16 (8.51–74.44)	<0.001	22.68 (7.37–69.82)	<0.001
4	Workload Sharing / Reducing Burden	Yes	Non-high	528	81	1.00		1.00	
		No	Non-high	394	139	3.01 (2.20–4.12)	<0.001	2.97 (2.13–4.14)	<0.001
		Yes	High	39	21	6.44 (3.29–12.61)	<0.001	5.65 (2.76–11.56)	<0.001
		No	High	39	31	21.38 (9.49–48.19)	<0.001	19.82 (8.47–46.4)	<0.001
5	Dedicated Space for Focused Work	Yes	Non-high	370	56	1.00		1.00	
		No	Non-high	552	164	2.37 (1.69–3.32)	<0.001	2.35 (1.64–3.36)	<0.001
		Yes	High	28	15	6.47 (2.92–14.33)	<0.001	5.40 (2.29–12.70)	<0.001
		No	High	50	37	15.96 (7.98–31.91)	<0.001	14.38 (6.95–29.78)	<0.001
6	Clear and Specific Work Instructions	Yes	Non-high	556	84	1.00		1.00	
		No	Non-high	366	136	3.32 (2.43–4.55)	<0.001	3.71 (2.64–5.20)	<0.001
		Yes	High	48	28	7.87 (4.24–14.61)	<0.001	6.87 (3.53–13.37)	<0.001
		No	High	30	24	22.48 (8.92–56.64)	<0.001	22.88 (8.63–60.65)	<0.001
7	Multiple Modes of Instruction Delivery	Yes	Non-high	533	88	1.00		1.00	
		No	Non-high	389	132	2.60 (1.90–3.54)	<0.001	2.40 (1.73–3.33)	<0.001
		Yes	High	38	23	7.75 (3.89–15.45)	<0.001	7.30 (3.50–15.21)	<0.001
		No	High	40	29	13.33 (6.42–27.68)	<0.001	10.51 (4.89–22.59)	<0.001
8	Advance Notice of Schedule Changes	Yes	Non-high	494	77	1.00		1.00	
		No	Non-high	428	143	2.72 (1.98–3.73)	<0.001	2.74 (1.96–3.84)	<0.001
		Yes	High	36	18	5.42 (2.70–10.87)	<0.001	4.48 (2.13–9.40)	<0.001
		No	High	42	34	23.02 (10.26–51.62)	<0.001	21.48 (9.22–50.02)	<0.001
9	Consideration for Task Focus	Yes	Non-high	457	78	1.00		1.00	
		No	Non-high	465	142	2.14 (1.56–2.92)	<0.001	2.22 (1.58–3.11)	<0.001
		Yes	High	34	18	5.47 (2.67–11.19)	<0.001	4.27 (1.98–9.21)	<0.001
		No	High	44	34	16.52 (7.84–34.83)	<0.001	16.58 (7.57–36.33)	<0.001
10	Regular Meetings with Supervisors	Yes	Non-high	479	66	1.00		1.00	
		No	Non-high	443	154	3.33 (2.41–4.62)	<0.001	3.07 (2.19–4.32)	<0.001
		Yes	High	39	21	7.30 (3.70–14.43)	<0.001	6.53 (3.19–13.38)	<0.001
		No	High	39	31	24.25 (10.69–55.03)	<0.001	19.87 (8.42–46.87)	<0.001
11	Task Assignment Considering Individual Strengths	Yes	Non-high	488	74	1.00		1.00	
		No	Non-high	434	146	2.84 (2.07–3.90)	<0.001	2.91 (2.08–4.07)	<0.001
		Yes	High	27	12	4.48 (2.01–9.95)	<0.001	3.94 (1.68–9.24)	0.002
		No	High	51	40	20.34 (9.99–41.45)	<0.001	18.27 (8.67–38.47)	<0.001
12	Consideration for Sensory Stimuli	Yes	Non-high	490	74	1.00		1.00	
		No	Non-high	432	146	2.87 (2.09–3.94)	<0.001	2.92 (2.09–4.08)	<0.001
		Yes	High	33	18	6.75 (3.26–13.98)	<0.001	6.32 (2.90–13.77)	<0.001
		No	High	45	34	17.38 (8.43–35.82)	<0.001	15.11 (7.09–32.24)	<0.001
13	Active Workplace Communication	Yes	Non-high	584	87	1.00		1.00	
		No	Non-high	338	133	3.71 (2.70–5.08)	<0.001	3.68 (2.63–5.14)	<0.001
		Yes	High	39	21	6.67 (3.41–13.02)	<0.001	5.36 (2.63–10.93)	<0.001
		No	High	39	31	22.14 (9.85–49.76)	<0.001	21.24 (9.06–49.81)	<0.001
14	Training on Diversity and Disability Awareness	Yes	Non-high	394	65	1.00		1.00	
		No	Non-high	528	155	2.10 (1.52–2.91)	<0.001	1.85 (1.31–2.60)	<0.001
		Yes	High	25	12	4.67 (2.04–10.70)	<0.001	4.41 (1.84–10.58)	0.001
		No	High	53	40	15.57 (7.89–30.74)	<0.001	11.80 (5.80–24.03)	<0.001

**(B) ADHD traits**									

**No.**	**Workplace adjustments**		**ADHD** **traits**	**No. of** **subjects**	**No. of** **cases**	**Univariate**		**Multivariable**	
	
	**Type**	**Presence**				**OR (95% CI)**	**p-value**	**OR (95% CI)**	**p-value**
1	Adjustment of Break Times	Yes	Non-high	540	94	1.00		1.00	
		No	Non-high	362	127	2.56 (1.88–3.50)	<0.001	2.60 (1.86–3.62)	<0.001
		Yes	High	42	16	2.92 (1.51–5.66)	0.001	3.46 (1.68–7.15)	0.001
		No	High	56	35	7.91 (4.41–14.19)	<0.001	8.45 (4.51–15.83)	<0.001
2	Quiet Rest Area	Yes	Non-high	476	88	1.00		1.00	
		No	Non-high	426	133	2.00 (1.47–2.73)	<0.001	2.08 (1.50–2.88)	<0.001
		Yes	High	39	11	1.73 (0.83–3.61)	0.143	1.84 (0.83–4.07)	0.135
		No	High	59	40	9.28 (5.13–16.80)	<0.001	10.85 (5.70–20.67)	<0.001
3	Consideration for Health / Flexible Work Hours	Yes	Non-high	626	121	1.00		1.00	
		No	Non-high	276	100	2.37 (1.73–3.25)	<0.001	2.40 (1.71–3.36)	<0.001
		Yes	High	56	22	2.70 (1.52–4.78)	<0.001	3.07 (1.66–5.68)	<0.001
		No	High	42	29	9.31 (4.70–18.44)	<0.001	10.96 (5.15–23.3)	<0.001
4	Workload Sharing / Reducing Burden	Yes	Non-high	532	89	1.00		1.00	
		No	Non-high	370	132	2.76 (2.02–3.77)	<0.001	2.67 (1.92–3.72)	<0.001
		Yes	High	35	13	2.94 (1.43–6.06)	0.003	3.12 (1.41–6.91)	0.005
		No	High	63	38	7.57 (4.35–13.16)	<0.001	8.44 (4.65–15.29)	<0.001
5	Dedicated Space for Focused Work	Yes	Non-high	371	61	1.00		1.00	
		No	Non-high	531	160	2.19 (1.57–3.05)	<0.001	2.12 (1.49–3.02)	<0.001
		Yes	High	27	10	2.99 (1.31–6.84)	0.010	2.77 (1.11–6.92)	0.030
		No	High	71	41	6.95 (4.03–11.98)	<0.001	7.92 (4.39–14.27)	<0.001
6	Clear and Specific Work Instructions	Yes	Non-high	555	90	1.00		1.00	
		No	Non-high	347	131	3.13 (2.29–4.29)	<0.001	3.45 (2.46–4.83)	<0.001
		Yes	High	49	22	4.21 (2.30–7.72)	<0.001	4.21 (2.15–8.23)	<0.001
		No	High	49	29	7.49 (4.06–13.83)	<0.001	10.36 (5.29–20.28)	<0.001
7	Multiple Modes of Instruction Delivery	Yes	Non-high	530	95	1.00		1.00	
		No	Non-high	372	126	2.35 (1.72–3.19)	<0.001	2.14 (1.54–2.97)	<0.001
		Yes	High	41	16	2.93 (1.51–5.70)	0.002	3.50 (1.71–7.13)	0.001
		No	High	57	35	7.29 (4.09–12.98)	<0.001	7.18 (3.84–13.41)	<0.001
8	Advance Notice of Schedule Changes	Yes	Non-high	495	79	1.00		1.00	
		No	Non-high	407	142	2.82 (2.06–3.87)	<0.001	2.85 (2.03–4.00)	<0.001
		Yes	High	35	16	4.43 (2.19–8.99)	<0.001	4.70 (2.16–10.22)	<0.001
		No	High	63	35	6.58 (3.79–11.43)	<0.001	7.78 (4.26–14.2)	<0.001
9	Consideration for Task Focus	Yes	Non-high	455	83	1.00		1.00	
		No	Non-high	447	138	2.00 (1.47–2.73)	<0.001	2.08 (1.49–2.92)	<0.001
		Yes	High	36	13	2.53 (1.23–5.21)	0.011	2.54 (1.15–5.60)	0.021
		No	High	62	38	7.10 (4.04–12.47)	<0.001	8.53 (4.64–15.71)	<0.001
10	Regular Meetings with Supervisors	Yes	Non-high	476	73	1.00		1.00	
		No	Non-high	426	148	2.94 (2.14–4.04)	<0.001	2.65 (1.89–3.71)	<0.001
		Yes	High	42	14	2.76 (1.39–5.49)	0.004	2.88 (1.38–6.00)	0.005
		No	High	56	37	10.75 (5.86–19.72)	<0.001	11.48 (5.96–22.11)	<0.001
11	Task Assignment Considering Individual Strengths	Yes	Non-high	484	75	1.00		1.00	
		No	Non-high	418	146	2.93 (2.13–4.02)	<0.001	2.91 (2.08–4.08)	<0.001
		Yes	High	31	11	3.00 (1.38–6.52)	0.006	2.95 (1.24–6.99)	0.014
		No	High	67	40	8.08 (4.68–13.96)	<0.001	9.28 (5.15–16.73)	<0.001
12	Consideration for Sensory Stimuli	Yes	Non-high	490	80	1.00		1.00	
		No	Non-high	412	141	2.67 (1.95–3.65)	<0.001	2.61 (1.87–3.65)	<0.001
		Yes	High	33	12	2.93 (1.39–6.19)	0.005	2.83 (1.26–6.37)	0.012
		No	High	65	39	7.69 (4.43–13.34)	<0.001	9.06 (4.97–16.51)	<0.001
13	Active Workplace Communication	Yes	Non-high	580	95	1.00		1.00	
		No	Non-high	322	126	3.28 (2.40–4.49)	<0.001	3.32 (2.37–4.64)	<0.001
		Yes	High	43	13	2.21 (1.11–4.40)	0.023	2.50 (1.20–5.21)	0.015
		No	High	55	38	11.41 (6.18–21.06)	<0.001	13.24 (6.77–25.88)	<0.001
14	Training on Diversity and Disability Awareness	Yes	Non-high	387	66	1.00		1.00	
		No	Non-high	515	155	2.09 (1.51–2.90)	<0.001	1.82 (1.29–2.57)	0.001
		Yes	High	32	11	2.55 (1.17–5.54)	0.018	3.28 (1.43–7.52)	0.005
		No	High	66	40	7.48 (4.27–13.1)	<0.001	6.71 (3.69–12.21)	<0.001

ASD: autism spectrum disorder; ADHD: attention-deficit/hyperactivity disorder; OR: odds ratio; CI: confidence interval; High: AQ ≥ 33 (ASD traits)/ ASRS ≥ 4 (ADHD traits); Non-high: AQ < 33 (ASD traits)/ ASRS < 4 (ADHD traits); No. of cases: Number of individuals with workplace maladjustment; Logistic regression analysis was conducted to estimate the ORs and 95% CIs for workplace maladjustment. The multivariable-adjusted model was adjusted for age, sex, marital status, annual household income, educational attainment, job type, occupational position, smoking status, drinking frequency, and leisure-time physical activity.

Supplementary Table 2 shows the stratified analyses of the associations of ASD and ADHD traits with workplace maladjustment by employment status and sex. This table presents the results of stratified analyses of Table 3 by employment status and sex. Stratified analyses by employment status and sex yielded results that were generally consistent with the main analyses. Among regular employees, the associations between ASD traits, ADHD traits, workplace adjustments, and workplace maladjustment showed similar directions and magnitudes to those observed in the overall sample. Similar patterns were also observed among non-regular employees. Likewise, stratified analyses by sex demonstrated that the associations were comparable in both males and females, with no substantial deviations from the primary findings. These results suggest that the main associations were not explained by differences in employment status or sex.

Table 4 presents the perceived necessity of reasonable accommodation–equivalent workplace adjustments among employees with high versus non-high ASD or ADHD traits. Workers with high ASD trait scores perceived such adjustments as strongly necessary more frequently than those with non-high scores for only 3 of the 14 adjustment types: multiple modes of instruction delivery (non-high ASD: 9.8%, high ASD: 19.2%; p = 0.030), task assignment considering individual strengths (9.9% vs. 19.2%; p = 0.035), and consideration for sensory stimuli (10.7% vs. 19.2%; p = 0.040). By contrast, workers with high ADHD trait scores perceived almost all types of reasonable accommodation–equivalent workplace adjustments as more strongly necessary than those with non-high scores, except for one item (regular meetings with supervisors).

**Table 4:** Perceived necessity of reasonable accommodation–equivalent workplace adjustments.

**(A) ASD traits**						

**No.**	**Workplace adjustments**	**ASD traits**	**Strongly necessary**	**Necessary**	**Other**	**p-value**
1	Adjustment of Break Times	Non-high	82 (8.9)	287 (31.1)	553 (60.0)	0.193
		High	11 (14.1)	27 (34.6)	40 (51.3)	
2	Quiet Rest Area	Non-high	119 (12.9)	343 (37.2)	460 (49.9)	0.141
		High	16 (20.5)	29 (37.2)	33 (42.3)	
3	Consideration for Health / Flexible Work Hours	Non-high	172 (18.7)	351 (38.1)	399 (43.3)	0.196
		High	21 (26.9)	28 (35.9)	29 (37.2)	
4	Workload Sharing / Reducing Burden	Non-high	120 (13.0)	351 (38.1)	451 (48.9)	0.084
		High	12 (15.4)	38 (48.7)	28 (35.9)	
5	Dedicated Space for Focused Work	Non-high	88 (9.5)	278 (30.2)	556 (60.3)	0.077
		High	13 (16.7)	26 (33.3)	39 (50.0)	
6	Clear and Specific Work Instructions	Non-high	130 (14.1)	341 (37.0)	451 (48.9)	0.052
		High	14 (17.9)	37 (47.4)	27 (34.6)	
7	Multiple Modes of Instruction Delivery	Non-high	90 (9.8)	349 (37.9)	483 (52.4)	0.030
		High	15 (19.2)	28 (35.9)	35 (44.9)	
8	Advance Notice of Schedule Changes	Non-high	122 (13.2)	366 (39.7)	434 (47.1)	0.053
		High	17 (21.8)	33 (42.3)	28 (35.9)	
9	Consideration for Task Focu	Non-high	85 (9.2)	312 (33.8)	525 (56.9)	0.169
		High	10 (12.8)	32 (41)	36 (46.2)	
10	Regular Meetings with Supervisors	Non-high	71 (7.7)	302 (32.8)	549 (59.5)	0.420
		High	6 (7.7)	20 (25.6)	52 (66.7)	
11	Task Assignment Considering Individual Strengths	Non-high	91 (9.9)	346 (37.5)	485 (52.6)	0.035
		High	15 (19.2)	27 (34.6)	36 (46.2)	
12	Consideration for Sensory Stimuli	Non-high	99 (10.7)	316 (34.3)	507 (55)	0.040
		High	15 (19.2)	29 (37.2)	34 (43.6)	
13	Active Workplace Communication	Non-high	116 (12.6)	341 (37)	465 (50.4)	0.632
		High	7 (9.0)	31 (39.7)	40 (51.3)	
14	Training on Diversity and Disability Awareness	Non-high	62 (6.7)	264 (28.6)	596 (64.6)	0.684
		High	7 (9.0)	20 (25.6)	51 (65.4)	

**(B) ADHD traits**						

**No.**	**Workplace adjustments**	**ADHD traits**	**Strongly necessary**	**Necessary**	**Other**	**p-value**
1	Adjustment of Break Times	Non-high	70 (7.8)	286 (31.7)	546 (60.5)	<0.001
		High	23 (23.5)	28 (28.6)	47 (48)	
2	Quiet Rest Area	Non-high	107 (11.9)	336 (37.3)	459 (50.9)	<0.001
		High	28 (28.6)	36 (36.7)	34 (34.7)	
3	Consideration for Health / Flexible Work Hours	Non-high	160 (17.7)	347 (38.5)	395 (43.8)	0.001
		High	33 (33.7)	32 (32.7)	33 (33.7)	
4	Workload Sharing / Reducing Burden	Non-high	106 (11.8)	351 (38.9)	445 (49.3)	<0.001
		High	26 (26.5)	38 (38.8)	34 (34.7)	
5	Dedicated Space for Focused Work	Non-high	77 (8.5)	278 (30.8)	547 (60.6)	<0.001
		High	24 (24.5)	26 (26.5)	48 (49)	
6	Clear and Specific Work Instructions	Non-high	117 (13.0)	341 (37.8)	444 (49.2)	<0.001
		High	27 (27.6)	37 (37.8)	34 (34.7)	
7	Multiple Modes of Instruction Delivery	Non-high	86 (9.5)	340 (37.7)	476 (52.8)	0.007
		High	19 (19.4)	37 (37.8)	42 (42.9)	
8	Advance Notice of Schedule Changes	Non-high	112 (12.4)	358 (39.7)	432 (47.9)	<0.001
		High	27 (27.6)	41 (41.8)	30 (30.6)	
9	Consideration for Task Focu	Non-high	75 (8.3)	308 (34.1)	519 (57.5)	<0.001
		High	20 (20.4)	36 (36.7)	42 (42.9)	
10	Regular Meetings with Supervisors	Non-high	65 (7.2)	289 (32.0)	548 (60.8)	0.162
		High	12 (12.2)	33 (33.7)	53 (54.1)	
11	Task Assignment Considering Individual Strengths	Non-high	87 (9.6)	328 (36.4)	487 (54)	<0.001
		High	19 (19.4)	45 (45.9)	34 (34.7)	
12	Consideration for Sensory Stimuli	Non-high	93 (10.3)	316 (35)	493 (54.7)	0.004
		High	21 (21.4)	29 (29.6)	48 (49)	
13	Active Workplace Communication	Non-high	103 (11.4)	339 (37.6)	460 (51)	0.036
		High	20 (20.4)	33 (33.7)	45 (45.9)	
14	Training on Diversity and Disability Awareness	Non-high	56 (6.2)	254 (28.2)	592 (65.6)	0.020
		High	13 (13.3)	30 (30.6)	55 (56.1)	

n (%); χ^2^-test; ASD: autism spectrum disorder; ADHD: attention-deficit/hyperactivity disorder; High: AQ ≥ 33 (ASD traits)/ ASRS ≥ 4 (ADHD traits); Non-high: AQ < 33 (ASD traits)/ ASRS < 4 (ADHD traits).

## Discussion

In this study, we examined the associations of ASD or ADHD traits and reasonable accommodation–equivalent workplace adjustments with workplace maladjustment. Even among workers with non-high ASD or ADHD trait scores, the odds of workplace maladjustment were significantly higher in workplaces without reasonable accommodation–equivalent adjustments than in those with such adjustments. Workers with high ASD or ADHD trait scores showed higher odds of workplace maladjustment than those with non-high trait scores, regardless of the presence or absence of reasonable accommodation–equivalent adjustments. Furthermore, among workers with high ASD or ADHD trait scores, the odds of workplace maladjustment were higher in the absence than in the presence of reasonable accommodation–equivalent adjustments. Regarding the perceived necessity of reasonable accommodation–equivalent workplace adjustments, most adjustments were perceived as equally necessary by workers with high and non-high ASD trait scores. By contrast, workers with high ADHD trait scores tended to perceive such adjustments as more strongly necessary than those with non-high ADHD trait scores.

Workers with high ASD or ADHD trait scores exhibited higher odds of workplace maladjustment than those with lower trait scores, irrespective of reasonable accommodation–equivalent adjustments. Moreover, among workers with high trait scores, maladjustment odds were substantially elevated in workplaces lacking such adjustments compared with those providing them. Previous case studies have reported the potential effectiveness of reasonable accommodations for workers with ASD and ADHD. The present cross-sectional study provides quantitative evidence suggesting an association between similar adjustments and better job adaptation among workers exhibiting high levels of ASD and ADHD traits.

Past reports have identified effective workplace accommodations for autistic employees, including consistent schedules and role clarity, task-organizing tools, reduced unstructured time, clear/direct communication with reminders and reassurance, organizational and coworker support, supportive leadership, advance notice of tasks, opportunities for breaks, appropriate positive feedback, and targeted environmental modifications (e.g., lighting adjustments, headphones, and seating to facilitate interaction) [28, 29]. For employees with ADHD, beneficial measures emphasize quiet or private workspaces, flexible hours, frequent breaks in long meetings, redistribution of tedious tasks, and broad use of assistive tools to support attention, memory, and organization (e.g., to-do lists, electronic planners/calendars, alarms/timers, reminders, note-taking systems, spellcheckers, templates, noise-canceling headphones, fidgets, and key/object finders) [14].

In the present study, the odds of maladjustment were higher among individuals with high levels of ASD traits than among those with high levels of ADHD traits. Previous register-based research has shown that, among young adults with neurodevelopmental disorders, ASDs are associated with the greatest burden of work incapacity, exceeding that observed for ADHD and learning disabilities [30], suggesting that, compared with individuals with ADHD, those with ASD may be particularly vulnerable to workplace maladjustment.

By contrast, across the two trait-based classifications, the patterns observed for reasonable accommodation–equivalent adjustments showed little difference. Although certain adjustments—for example, considerations for sensory stimuli [28]—are often regarded as particularly relevant to ASD, our findings demonstrate comparable associations among individuals with high levels of ADHD traits. This may indicate that workplaces implementing such adjustments cultivate a generally supportive environment that facilitates adaptation, even when the specific modifications are not directly aligned with an individual’s predominant neurodevelopmental traits.

Among workers with lower ASD or ADHD trait scores, those in workplaces lacking reasonable accommodation–equivalent adjustments showed significantly higher odds of workplace maladjustment compared with those in workplaces offering such adjustments. These cross-sectional findings suggest that such adjustments may reduce maladjustment risk even among workers without pronounced ASD or ADHD traits, potentially creating more comfortable and supportive environments for the broader workforce. This aligns with universal design [18] principles, which posit that accommodations for employees with developmental disabilities also benefit those without. Previous studies support this, showing that implementing universal design in facilities enhances human performance, health, wellness, and social participation [31]. The findings from our study empirically support the concept that workplace environments designed with inclusivity principles can enhance well-being and retention across diverse employee groups, reinforcing the value of universal design in organizational health promotion. It remains unclear why workers with lower ASD or ADHD trait scores showed a similar but weaker pattern than those with high trait scores. Therefore, future longitudinal and intervention studies are needed to clarify the underlying mechanisms. These results also suggest that workplace adjustments equivalent to reasonable accommodations may be beneficial not only for employees with pronounced neurodevelopmental traits but also for those with milder or subclinical traits, which supports the broader applicability of inclusive workplace practices.

The stratified analyses by employment status and sex supported the robustness of our main findings. Because employment structure and work style differ substantially across regular and non-regular employees in Japan, and because the meaning of workplace adjustments may vary by sex, these factors could potentially confound the associations examined in this study. However, the stratified analyses showed generally consistent associations between ASD and ADHD traits, workplace adjustments, and workplace maladjustment across employment status and between males and females. These findings suggest that the main results were not materially driven by employment structure or sex, although residual confounding cannot be completely ruled out.

Regarding the perceived necessity of reasonable accommodation–equivalent workplace adjustments, most adjustments were perceived as equally necessary by workers with high and non-high ASD trait scores, contrary to our hypothesis that workers with high ASD trait scores would perceive a stronger need for each workplace adjustment than those with non-high ASD trait scores. In previous research, participants with ASD had significantly more emotional self-awareness difficulties than those without ASD [32]. Such findings suggest that individuals with high levels of ASD traits may struggle to accurately appraise their emotional strain and workplace difficulties. Therefore, the lack of a stronger perceived need for ASD-related adjustments among workers with high ASD trait scores in our study may indicate limited awareness of their own difficulties and support needs rather than a genuinely lower requirement for such adjustments. These findings thus highlight the importance of awareness and proactive support from colleagues, supervisors, and others in the workplace.

Notably, in our study, workers with high ADHD trait scores tended to perceive such adjustments as more strongly necessary than those with non-high ADHD trait scores, because workers with high ADHD traits may be more aware of their need for such adjustments and therefore more likely to initiate requests for support.

It should be noted that workplace maladjustment in this study should not be interpreted as an objective risk factor in itself. Rather, it reflects workers’ subjective perceptions of their workplace environment and the extent to which they perceive a need for support or interventions. Therefore, our findings should be understood as indicating how workers with different ASD or ADHD trait levels perceive their work environment and the potential relevance of workplace adjustments, rather than as evidence that workplace maladjustment objectively exists as a workplace characteristic.

### Limitations

This study has several limitations that should be acknowledged. First, because of its cross-sectional design, causal relationships between ASD or ADHD traits, workplace adjustments, and workplace maladjustment cannot be determined. Second, the information about ASD or ADHD traits, workplace adjustments, and perceived necessity was based on self-reported data, which may be subject to reporting bias or inaccuracies. Third, although participants were randomly selected from a nationwide online panel of Japanese workers, generalisability may be limited to populations with limited Internet access or lower digital literacy. Future research should adopt longitudinal or intervention designs to elucidate the causal mechanisms underlying these associations and to assess the long-term effects of workplace adjustments equivalent to reasonable accommodations—those enhancing employees’ workplace adaptation—on well-being and job retention. Fourth, although the SASS has been validated as a reliable indicator of general social functioning, it does not specifically assess workplace-related aspects of adjustment, such as relationships with supervisors, job satisfaction, or work-specific stressors. Therefore, the use of SASS may not fully capture workplace-specific maladjustment. Fifth, because stratified analyses inherently reduce the sample size within each subgroup, the statistical precision was lower than in the main analyses. In particular, the number of non-regular employees was relatively small, and the estimates for this subgroup should be interpreted with caution.

## Conclusions

The present findings from this cross-sectional study quantitatively suggest that workplace adjustments equivalent to reasonable accommodations are associated with a lower risk of workplace maladjustment—and consequently with greater workplace adaptation—among employees with high ASD or ADHD trait levels. This underscores the critical importance of providing such support to promote their adjustment and continued employment.

## Abbreviations

ADHD: Attention-deficit/hyperactivity disorder
AQ: Autism-Spectrum Quotient
ASD: Autism spectrum disorder
ASRS: adult ADHD Self-Report Scale
CI: confidence interval
CRPD: Convention on the Rights of Persons with Disabilities
OR: odds ratio
SASS: Social Adaptation Self-evaluation Scale

## Supplementary information

**Additional file 1:** **Supplementary Fig. 1.** Participant flowchart. **Supplementary Table 1.** Abbreviations and corresponding questionnaire items used in the study. **Supplementary Table 2.** Stratified associations of ASD and ADHD traits with workplace maladjustment by employment status and sex.

## Declarations

### Availability of data and materials

Data available on request from authors.

### Generative AI and AI-assisted technologies in the writing process

During the preparation of this manuscript, the authors used OpenAI’s ChatGPT and Perplexity AI to improve language and readability. After using these tools, the authors reviewed and edited the content and take full responsibility for the final version of the publication. No generative AI tools were used in data analysis, data interpretation, or table generation.
