# Supplementary material for: Workplace adjustments equivalent to reasonable accommodations and workplace maladjustment in a general working population, with a focus on high autism spectrum disorder (ASD) and attention deficit/hyperactivity disorder (ADHD) traits
Source: Environ Health Prev Med. 2026 Sep 17;31:62. doi: 10.1265/ehpm.26-00080 (PMC13617183; doi:10.1265/ehpm.26-00080)
Supplement: Supplementary file 1 [file ehpm-31-062-s001.docx]

**Registered Panel Members**

(Rakuten Insight Inc., Tokyo, Japan)

n = 279,808 across all Japanese prefectures

**Randomly selected and invited to participate**

n = 6,624

　───► **Excluded: Non-respondents**

n = 4,279

**Responded to Screening**

n = 2,345

───► **Excluded: Did not meet eligibility criteria**

n = 1,040

• Aged outside 20–59 years

• Not currently employed

**Eligible for Study / Granted Access**

Aged 20–59 years and currently employed

n = 1,305

**Completed the self-administered questionnaire**

n = 1,305

No missing data were present

because the survey system required a response for each item before submission.

**Random Selection by Survey Company**

n = 1,000

**Final Analytic Sample**

n = 1,000

Balanced by sex (1:1 ratio) and

age groups (equal numbers across 20–29, 30–39, 40–49, and 50–59 years)

**Supplementary Fig.1. Participant flowchart.**

Supplementary Table 1. Abbreviations and corresponding questionnaire items used in the study

English items

|  | Reasonable Accommodation–Equivalent | |
| --- | --- | --- |
|  | Abbreviation | Questionnaire items |
| 1 | Adjustment of Break Times | Is it possible to adjust break times according to fatigue level or individual needs? |
| 2 | Quiet Rest Area | Is there a space where you can take a quiet break? |
| 3 | Consideration for Health / Flexible Work Hours | Is it easy to take leave or adjust working hours when you feel unwell or need to attend medical appointments? |
| 4 | Workload Sharing / Reducing Burden | Is it possible to share tasks or adjust break times to reduce workload? |
| 5 | Dedicated Space for Focused Work | Is there an individual space where you can work with minimal distractions? |
| 6 | Clear and Specific Work Instructions | Are work instructions provided clearly and specifically? |
| 7 | Multiple Modes of Instruction Delivery | Can work instructions be confirmed not only verbally but also in writing or via digital tools? |
| 8 | Advance Notice of Schedule Changes | Are changes to work schedules communicated well in advance? |
| 9 | Consideration for Task Focus | Are you generally given one task at a time so you can concentrate without being instructed to handle multiple tasks simultaneously? |
| 10 | Regular Meetings with Supervisors | Do you have regular meetings or consultation opportunities with your supervisor? |
| 11 | Task Assignment Considering Individual Strengths | Are job assignments made with consideration of individual characteristics and strengths? |
| 12 | Consideration for Sensory Stimuli | Does the workplace provide accommodations for noise, lighting, or visual stimuli (e.g., quiet spaces, adjusted lighting)? |
| 13 | Active Workplace Communication | Is communication in the workplace active, such as checking in with each other or offering assistance? |
| 14 | Training on Diversity and Disability Awareness | Are there training sessions or briefings to promote understanding of workplace diversity and disabilities? |

Japanese original items

|  | 質問項目 |
| --- | --- |
| 1 | 疲労度や個人の状況に応じて、休憩時間の調整が可能ですか？ |
| 2 | 静かに休憩できるスペースがありますか？ |
| 3 | 体調不良時や通院での休暇取得や勤務時間の調整はしやすいですか？ |
| 4 | 作業負担を軽減するため、業務の分担や休憩時間の調整が可能ですか？ |
| 5 | 集中して作業できる個別のスペースがありますか？ |
| 6 | 業務指示は明確で具体的に行われていますか？ |
| 7 | 業務指示は口頭だけでなく、書面やデジタルツールでも確認できますか？ |
| 8 | 業務スケジュールの変更は、十分な余裕を持って伝えられていますか？ |
| 9 | 一度に複数の作業を指示されることは少なく、一度に一つの作業に集中できるよう配慮されていますか？ |
| 10 | 上司との定期的な面談や相談の機会がありますか？ |
| 11 | 個人の特性や得意分野を考慮した業務分担や配置が行われていますか？ |
| 12 | 職場環境において、騒音や照明、視覚的な刺激に対する配慮（静かな場所の提供、照明の調整など）が行われていますか？ |
| 13 | 職場では、お互いに確認し合ったり、声を掛け合ったりするコミュニケーションが活発ですか？ |
| 14 | 職場の多様性や障害の理解を深めるための研修や説明会が行われていますか？ |

Supplementary Table 2. Stratified associations of ASD and ADHD traits with workplace maladjustment by employment status and sex.

Employment status (A) ASD traits

| No. | Workplace adjustments | | | ASD  traits | No. of subjects | No. of cases | Univariate | | Multivariable | |
| --- | --- | --- | --- | --- | --- | --- | --- | --- | --- | --- |
|  | Type | Employment status | Presence |  |  |  | OR (95% CI) | p-value | OR (95% CI) | p-value |
| 1 | Adjustment of Break Times | Regular | Yes | Non-high | 387 | 58 | 1.00 |  | 1.00 |  |
|  |  |  | No | Non-high | 279 | 92 | 2.79 (1.92-4.06) | <0.001 | 2.70 (1.81-4.04) | <0.001 |
|  |  |  | Yes | High | 23 | 13 | 7.37 (3.09-17.61) | <0.001 | 6.73 (2.64-17.18) | <0.001 |
|  |  |  | No | High | 28 | 22 | 20.80 (8.09-53.50) | <0.001 | 17.08 (6.29-46.39) | <0.001 |
|  |  | Non-regular | Yes | Non-high | 115 | 19 | 1.00 |  | 1.00 |  |
|  |  |  | No | Non-high | 84 | 29 | 2.66 (1.37-5.19) | 0.004 | 2.96 (1.42-6.18) | 0.004 |
|  |  |  | Yes | High | 12 | 7 | 7.07 (2.03-24.66) | 0.002 | 6.84 (1.81-25.85) | 0.005 |
|  |  |  | No | High | 9 | 6 | 10.11 (2.32-43.98) | 0.002 | 8.36 (1.70-41.08) | 0.009 |
| 2 | Quiet Rest Area | Regular | Yes | Non-high | 338 | 53 | 1.00 |  | 1.00 |  |
|  |  |  | No | Non-high | 328 | 97 | 2.26 (1.55-3.29) | <0.001 | 2.38 (1.59-3.56) | <0.001 |
|  |  |  | Yes | High | 17 | 8 | 4.78 (1.77-12.95) | 0.002 | 4.09 (1.41-11.89) | 0.010 |
|  |  |  | No | High | 34 | 27 | 20.74 (8.59-50.08) | <0.001 | 18.87 (7.42-47.97) | <0.001 |
|  |  | Non-regular | Yes | Non-high | 108 | 18 | 1.00 |  | 1.00 |  |
|  |  |  | No | Non-high | 91 | 30 | 2.46 (1.26-4.80) | 0.008 | 3.08 (1.44-6.55) | 0.004 |
|  |  |  | Yes | High | 12 | 7 | 7.00 (2.00-24.54) | 0.002 | 5.85 (1.48-23.19) | 0.012 |
|  |  |  | No | High | 9 | 6 | 10.00 (2.29-43.73) | 0.002 | 13.02 (2.54-66.71) | 0.002 |
| 3 | Consideration for Health / Flexible Work Hours | Regular | Yes | Non-high | 441 | 74 | 1.00 |  | 1.00 |  |
|  |  |  | No | Non-high | 225 | 76 | 2.53 (1.74-3.67) | <0.001 | 2.72 (1.82-4.08) | <0.001 |
|  |  |  | Yes | High | 33 | 18 | 5.95 (2.87-12.34) | <0.001 | 5.29 (2.39-11.71) | <0.001 |
|  |  |  | No | High | 18 | 17 | 84.31 (11.05-643.36) | <0.001 | 75.58 (9.58-596.36) | <0.001 |
|  |  | Non-regular | Yes | Non-high | 151 | 28 | 1.00 |  | 1.00 |  |
|  |  |  | No | Non-high | 48 | 20 | 3.14 (1.55-6.35) | 0.001 | 3.67 (1.66-8.11) | 0.001 |
|  |  |  | Yes | High | 17 | 10 | 6.28 (2.20-17.92) | 0.001 | 5.68 (1.82-17.72) | 0.003 |
|  |  |  | No | High | 4 | 3 | 13.18 (1.32-131.46) | 0.028 | 14.11 (1.21-164.58) | 0.035 |
| 4 | Workload Sharing / Reducing Burden | Regular | Yes | Non-high | 373 | 49 | 1.00 |  | 1.00 |  |
|  |  |  | No | Non-high | 293 | 101 | 3.48 (2.37-5.11) | <0.001 | 3.58 (2.37-5.40) | <0.001 |
|  |  |  | Yes | High | 24 | 13 | 7.81 (3.32-18.42) | <0.001 | 7.16 (2.87-17.84) | <0.001 |
|  |  |  | No | High | 27 | 22 | 29.09 (10.53-80.40) | <0.001 | 26.09 (8.88-76.64) | <0.001 |
|  |  | Non-regular | Yes | Non-high | 118 | 23 | 1.00 |  | 1.00 |  |
|  |  |  | No | Non-high | 81 | 25 | 1.84 (0.96-3.55) | 0.067 | 1.88 (0.92-3.82) | 0.082 |
|  |  |  | Yes | High | 13 | 7 | 4.82 (1.48-15.71) | 0.009 | 4.30 (1.19-15.58) | 0.026 |
|  |  |  | No | High | 8 | 6 | 12.39 (2.35-65.42) | 0.003 | 12.35 (2.09-72.96) | 0.006 |
| 5 | Dedicated Space for Focused Work | Regular | Yes | Non-high | 262 | 36 | 1.00 |  | 1.00 |  |
|  |  |  | No | Non-high | 404 | 114 | 2.47 (1.63-3.73) | <0.001 | 2.37 (1.53-3.66) | <0.001 |
|  |  |  | Yes | High | 15 | 7 | 5.49 (1.88-16.07) | 0.002 | 4.44 (1.41-13.91) | 0.011 |
|  |  |  | No | High | 36 | 28 | 21.97 (9.29-51.97) | <0.001 | 19.04 (7.65-47.42) | <0.001 |
|  |  | Non-regular | Yes | Non-high | 76 | 13 | 1.00 |  | 1.00 |  |
|  |  |  | No | Non-high | 123 | 35 | 1.93 (0.94-3.94) | 0.072 | 1.96 (0.91-4.20) | 0.085 |
|  |  |  | Yes | High | 9 | 6 | 9.69 (2.14-43.83) | 0.003 | 8.92 (1.72-46.36) | 0.009 |
|  |  |  | No | High | 12 | 7 | 6.79 (1.86-24.74) | 0.004 | 6.07 (1.48-24.81) | 0.012 |
| 6 | Clear and Specific Work Instructions | Regular | Yes | Non-high | 386 | 53 | 1.00 |  | 1.00 |  |
|  |  |  | No | Non-high | 280 | 97 | 3.33 (2.28-4.87) | <0.001 | 3.80 (2.52-5.74) | <0.001 |
|  |  |  | Yes | High | 29 | 18 | 10.28 (4.60-22.98) | <0.001 | 9.75 (4.06-23.40) | <0.001 |
|  |  |  | No | High | 22 | 17 | 21.36 (7.56-60.34) | <0.001 | 20.57 (6.79-62.36) | <0.001 |
|  |  | Non-regular | Yes | Non-high | 128 | 20 | 1.00 |  | 1.00 |  |
|  |  |  | No | Non-high | 71 | 28 | 3.52 (1.79-6.90) | <0.001 | 4.33 (1.99-9.41) | <0.001 |
|  |  |  | Yes | High | 16 | 8 | 5.40 (1.82-16.06) | 0.002 | 4.57 (1.41-14.82) | 0.011 |
|  |  |  | No | High | 5 | 5 | ­ | ­ | ­ | ­ |
| 7 | Multiple Modes of Instruction Delivery | Regular | Yes | Non-high | 398 | 60 | 1.00 |  | 1.00 |  |
|  |  |  | No | Non-high | 268 | 90 | 2.85 (1.96-4.14) | <0.001 | 2.80 (1.87-4.18) | <0.001 |
|  |  |  | Yes | High | 26 | 16 | 9.01 (3.91-20.80) | <0.001 | 8.84 (3.59-21.79) | <0.001 |
|  |  |  | No | High | 25 | 19 | 17.84 (6.84-46.50) | <0.001 | 13.78 (5.04-37.67) | <0.001 |
|  |  | Non-regular | Yes | Non-high | 109 | 21 | 1.00 |  | 1.00 |  |
|  |  |  | No | Non-high | 90 | 27 | 1.80 (0.93-3.46) | 0.080 | 1.84 (0.89-3.81) | 0.103 |
|  |  |  | Yes | High | 11 | 6 | 5.03 (1.40-18.06) | 0.013 | 5.09 (1.24-20.87) | 0.024 |
|  |  |  | No | High | 10 | 7 | 9.78 (2.33-41.02) | 0.002 | 7.97 (1.70-37.25) | 0.008 |
| 8 | Advance Notice of Schedule Changes | Regular | Yes | Non-high | 345 | 51 | 1.00 |  | 1.00 |  |
|  |  |  | No | Non-high | 321 | 99 | 2.57 (1.76-3.76) | <0.001 | 2.61 (1.74-3.90) | <0.001 |
|  |  |  | Yes | High | 18 | 9 | 5.77 (2.18-15.22) | <0.001 | 5.24 (1.87-14.69) | 0.002 |
|  |  |  | No | High | 33 | 26 | 21.41 (8.83-51.93) | <0.001 | 18.08 (7.11-45.99) | <0.001 |
|  |  | Non-regular | Yes | Non-high | 117 | 18 | 1.00 |  | 1.00 |  |
|  |  |  | No | Non-high | 82 | 30 | 3.17 (1.62-6.23) | 0.001 | 3.02 (1.42-6.42) | 0.004 |
|  |  |  | Yes | High | 15 | 7 | 4.81 (1.55-14.93) | 0.007 | 3.97 (1.19-13.29) | 0.025 |
|  |  |  | No | High | 6 | 6 | ­ | ­ | ­ | ­ |
| 9 | Consideration for Task Focus | Regular | Yes | Non-high | 304 | 47 | 1.00 |  | 1.00 |  |
|  |  |  | No | Non-high | 362 | 103 | 2.18 (1.48-3.20) | <0.001 | 2.29 (1.52-3.47) | <0.001 |
|  |  |  | Yes | High | 19 | 9 | 4.92 (1.90-12.76) | 0.001 | 4.57 (1.64-12.77) | 0.004 |
|  |  |  | No | High | 32 | 26 | 23.70 (9.25-60.69) | <0.001 | 20.88 (7.74-56.33) | <0.001 |
|  |  | Non-regular | Yes | Non-high | 117 | 22 | 1.00 |  | 1.00 |  |
|  |  |  | No | Non-high | 82 | 26 | 2.01 (1.04-3.87) | 0.038 | 2.03 (0.98-4.22) | 0.058 |
|  |  |  | Yes | High | 12 | 7 | 6.05 (1.75-20.85) | 0.004 | 4.98 (1.32-18.79) | 0.018 |
|  |  |  | No | High | 9 | 6 | 8.64 (2.00-37.24) | 0.004 | 9.46 (1.87-47.94) | 0.007 |
| 10 | Regular Meetings with Supervisors | Regular | Yes | Non-high | 370 | 52 | 1.00 |  | 1.00 |  |
|  |  |  | No | Non-high | 296 | 98 | 3.03 (2.07-4.43) | <0.001 | 2.69 (1.80-4.02) | <0.001 |
|  |  |  | Yes | High | 27 | 15 | 7.64 (3.39-17.25) | <0.001 | 6.71 (2.84-15.86) | <0.001 |
|  |  |  | No | High | 24 | 20 | 30.58 (10.05-93.04) | <0.001 | 23.46 (7.30-75.37) | <0.001 |
|  |  | Non-regular | Yes | Non-high | 89 | 10 | 1.00 |  | 1.00 |  |
|  |  |  | No | Non-high | 110 | 38 | 4.17 (1.94-8.97) | <0.001 | 6.06 (2.56-14.32) | <0.001 |
|  |  |  | Yes | High | 10 | 5 | 7.90 (1.94-32.14) | 0.004 | 11.36 (2.31-55.94) | 0.003 |
|  |  |  | No | High | 11 | 8 | 21.07 (4.79-92.64) | <0.001 | 19.58 (3.86-99.36) | <0.001 |
| 11 | Task Assignment Considering Individual Strengths | Regular | Yes | Non-high | 347 | 45 | 1.00 |  | 1.00 |  |
|  |  |  | No | Non-high | 319 | 105 | 3.29 (2.23-4.87) | <0.001 | 3.41 (2.25-5.15) | <0.001 |
|  |  |  | Yes | High | 15 | 7 | 5.87 (2.03-16.98) | 0.001 | 5.59 (1.80-17.40) | 0.003 |
|  |  |  | No | High | 36 | 28 | 23.49 (10.08-54.73) | <0.001 | 20.34 (8.28-49.96) | <0.001 |
|  |  | Non-regular | Yes | Non-high | 107 | 19 | 1.00 |  | 1.00 |  |
|  |  |  | No | Non-high | 92 | 29 | 2.13 (1.10-4.14) | 0.025 | 2.16 (1.06-4.40) | 0.034 |
|  |  |  | Yes | High | 10 | 4 | 3.09 (0.79-12.02) | 0.104 | 2.53 (0.59-10.89) | 0.213 |
|  |  |  | No | High | 11 | 9 | 20.84 (4.16-104.31) | <0.001 | 20.26 (3.69-111.19) | 0.001 |
| 12 | Consideration for Sensory Stimuli | Regular | Yes | Non-high | 351 | 48 | 1.00 |  | 1.00 |  |
|  |  |  | No | Non-high | 315 | 102 | 3.02 (2.06-4.44) | <0.001 | 3.19 (2.11-4.82) | <0.001 |
|  |  |  | Yes | High | 19 | 10 | 7.01 (2.71-18.15) | <0.001 | 7.40 (2.64-20.74) | <0.001 |
|  |  |  | No | High | 32 | 25 | 22.55 (9.24-54.99) | <0.001 | 18.16 (7.14-46.17) | <0.001 |
|  |  | Non-regular | Yes | Non-high | 105 | 17 | 1.00 |  | 1.00 |  |
|  |  |  | No | Non-high | 94 | 31 | 2.55 (1.30-5.00) | 0.007 | 3.09 (1.48-6.44) | 0.003 |
|  |  |  | Yes | High | 11 | 6 | 6.21 (1.70-22.69) | 0.006 | 6.76 (1.63-28.03) | 0.008 |
|  |  |  | No | High | 10 | 7 | 12.08 (2.84-51.43) | 0.001 | 10.11 (2.11-48.40) | 0.004 |
| 13 | Active Workplace Communication | Regular | Yes | Non-high | 415 | 59 | 1.00 |  | 1.00 |  |
|  |  |  | No | Non-high | 251 | 91 | 3.43 (2.35-5.00) | <0.001 | 3.65 (2.43-5.48) | <0.001 |
|  |  |  | Yes | High | 25 | 13 | 6.54 (2.85-15.01) | <0.001 | 5.75 (2.34-14.09) | <0.001 |
|  |  |  | No | High | 26 | 22 | 33.19 (11.04-99.74) | <0.001 | 31.14 (9.74-99.49) | <0.001 |
|  |  | Non-regular | Yes | Non-high | 138 | 22 | 1.00 |  | 1.00 |  |
|  |  |  | No | Non-high | 61 | 26 | 3.92 (1.98-7.75) | <0.001 | 4.82 (2.20-10.55) | <0.001 |
|  |  |  | Yes | High | 11 | 6 | 6.33 (1.78-22.56) | 0.004 | 5.20 (1.29-20.89) | 0.020 |
|  |  |  | No | High | 10 | 7 | 12.30 (2.95-51.27) | 0.001 | 11.54 (2.40-55.51) | 0.002 |
| 14 | Training on Diversity and Disability Awareness | Regular | Yes | Non-high | 315 | 52 | 1.00 |  | 1.00 |  |
|  |  |  | No | Non-high | 351 | 98 | 1.96 (1.34-2.86) | <0.001 | 1.74 (1.17-2.60) | 0.006 |
|  |  |  | Yes | High | 18 | 9 | 5.06 (1.92-13.35) | 0.001 | 4.45 (1.60-12.40) | 0.004 |
|  |  |  | No | High | 33 | 26 | 18.79 (7.75-45.56) | <0.001 | 14.51 (5.70-36.92) | <0.001 |
|  |  | Non-regular | Yes | Non-high | 64 | 10 | 1.00 |  | 1.00 |  |
|  |  |  | No | Non-high | 135 | 38 | 2.12 (0.98-4.58) | 0.057 | 2.22 (0.96-5.14) | 0.063 |
|  |  |  | Yes | High | 5 | 2 | 3.60 (0.53-24.37) | 0.189 | 3.98 (0.51-31.04) | 0.187 |
|  |  |  | No | High | 16 | 11 | 11.88 (3.39-41.64) | <0.001 | 10.16 (2.66-38.85) | 0.001 |

Employment status (B) ADHD traits

| No. | Workplace adjustments | | | ADHD  traits | No. of subjects | No. of cases | Univariate | | Multivariable | |
| --- | --- | --- | --- | --- | --- | --- | --- | --- | --- | --- |
|  | Type | Employment status | Presence |  |  |  | OR (95% CI) | p-value | OR (95% CI) | p-value |
| 1 | Adjustment of Break Times | Regular | Yes | Non-high | 378 | 59 | 1.00 |  | 1.00 |  |
|  |  |  | No | Non-high | 268 | 91 | 2.78 (1.91-4.05) | <0.001 | 2.68 (1.79-4.02) | <0.001 |
|  |  |  | Yes | High | 32 | 12 | 3.24 (1.51-6.99) | 0.003 | 4.51 (1.91-10.65) | 0.001 |
|  |  |  | No | High | 39 | 23 | 7.77 (3.88-15.59) | <0.001 | 8.19 (3.86-17.38) | <0.001 |
|  |  | Non-regular | Yes | Non-high | 120 | 23 | 1.00 |  | 1.00 |  |
|  |  |  | No | Non-high | 80 | 27 | 2.15 (1.12-4.11) | 0.021 | 2.35 (1.14-4.83) | 0.021 |
|  |  |  | Yes | High | 7 | 3 | 3.16 (0.66-15.12) | 0.149 | 7.89 (1.28-48.55) | 0.026 |
|  |  |  | No | High | 13 | 8 | 6.75 (2.02-22.55) | 0.002 | 9.96 (2.54-39.11) | 0.001 |
| 2 | Quiet Rest Area | Regular | Yes | Non-high | 327 | 53 | 1.00 |  | 1.00 |  |
|  |  |  | No | Non-high | 319 | 97 | 2.26 (1.55-3.30) | <0.001 | 2.41 (1.61-3.61) | <0.001 |
|  |  |  | Yes | High | 28 | 8 | 2.07 (0.87-4.94) | 0.102 | 2.63 (1.00-6.90) | 0.050 |
|  |  |  | No | High | 43 | 27 | 8.72 (4.40-17.3) | <0.001 | 11.1 (5.20-23.69) | <0.001 |
|  |  | Non-regular | Yes | Non-high | 111 | 23 | 1.00 |  | 1.00 |  |
|  |  |  | No | Non-high | 89 | 27 | 1.67 (0.88-3.17) | 0.120 | 2.32 (1.10-4.88) | 0.027 |
|  |  |  | Yes | High | 9 | 2 | 1.09 (0.21-5.62) | 0.915 | 1.57 (0.25-9.80) | 0.631 |
|  |  |  | No | High | 11 | 9 | 17.22 (3.48-85.23) | <0.001 | 63.58 (8.84-457.20) | <0.001 |
| 3 | Consideration for Health / Flexible Work Hours | Regular | Yes | Non-high | 435 | 79 | 1.00 |  | 1.00 |  |
|  |  |  | No | Non-high | 211 | 71 | 2.29 (1.57-3.33) | <0.001 | 2.42 (1.60-3.65) | <0.001 |
|  |  |  | Yes | High | 39 | 13 | 2.25 (1.11-4.58) | 0.025 | 2.64 (1.21-5.74) | 0.014 |
|  |  |  | No | High | 32 | 22 | 9.91 (4.52-21.76) | <0.001 | 14.74 (6.02-36.06) | <0.001 |
|  |  | Non-regular | Yes | Non-high | 154 | 31 | 1.00 |  | 1.00 |  |
|  |  |  | No | Non-high | 46 | 19 | 2.79 (1.38-5.66) | 0.004 | 3.34 (1.48-7.52) | 0.004 |
|  |  |  | Yes | High | 14 | 7 | 3.97 (1.30-12.15) | 0.016 | 6.92 (1.87-25.65) | 0.004 |
|  |  |  | No | High | 6 | 4 | 7.94 (1.39-45.32) | 0.020 | 20.68 (2.45-174.69) | 0.005 |
| 4 | Workload Sharing / Reducing Burden | Regular | Yes | Non-high | 371 | 54 | 1.00 |  | 1.00 |  |
|  |  |  | No | Non-high | 275 | 96 | 3.15 (2.15-4.61) | <0.001 | 3.16 (2.10-4.76) | <0.001 |
|  |  |  | Yes | High | 26 | 8 | 2.61 (1.08-6.30) | 0.033 | 3.23 (1.22-8.55) | 0.018 |
|  |  |  | No | High | 45 | 27 | 8.81 (4.54-17.08) | <0.001 | 10.95 (5.28-22.68) | <0.001 |
|  |  | Non-regular | Yes | Non-high | 124 | 26 | 1.00 |  | 1.00 |  |
|  |  |  | No | Non-high | 76 | 24 | 1.74 (0.91-3.33) | 0.094 | 1.67 (0.82-3.40) | 0.158 |
|  |  |  | Yes | High | 7 | 4 | 5.03 (1.06-23.87) | 0.042 | 7.68 (1.33-44.23) | 0.023 |
|  |  |  | No | High | 13 | 7 | 4.40 (1.36-14.21) | 0.013 | 8.00 (2.01-31.88) | 0.003 |
| 5 | Dedicated Space for Focused Work | Regular | Yes | Non-high | 257 | 37 | 1.00 |  | 1.00 |  |
|  |  |  | No | Non-high | 389 | 113 | 2.43 (1.61-3.67) | <0.001 | 2.32 (1.50-3.59) | <0.001 |
|  |  |  | Yes | High | 20 | 6 | 2.55 (0.92-7.05) | 0.072 | 3.10 (1.02-9.44) | 0.047 |
|  |  |  | No | High | 51 | 29 | 7.84 (4.07-15.08) | <0.001 | 9.22 (4.49-18.97) | <0.001 |
|  |  | Non-regular | Yes | Non-high | 82 | 17 | 1.00 |  | 1.00 |  |
|  |  |  | No | Non-high | 118 | 33 | 1.48 (0.76-2.90) | 0.247 | 1.42 (0.69-2.94) | 0.338 |
|  |  |  | Yes | High | 3 | 2 | 7.65 (0.65-89.43) | 0.105 | 12.95 (0.66-253.87) | 0.092 |
|  |  |  | No | High | 17 | 9 | 4.30 (1.44-12.82) | 0.009 | 7.42 (2.09-26.34) | 0.002 |
| 6 | Clear and Specific Work Instructions | Regular | Yes | Non-high | 381 | 57 | 1.00 |  | 1.00 |  |
|  |  |  | No | Non-high | 265 | 93 | 3.07 (2.11-4.49) | <0.001 | 3.41 (2.26-5.14) | <0.001 |
|  |  |  | Yes | High | 34 | 14 | 3.98 (1.90-8.33) | <0.001 | 4.64 (2.03-10.60) | <0.001 |
|  |  |  | No | High | 37 | 21 | 7.46 (3.67-15.16) | <0.001 | 11.25 (5.06-25.03) | <0.001 |
|  |  | Non-regular | Yes | Non-high | 134 | 23 | 1.00 |  | 1.00 |  |
|  |  |  | No | Non-high | 66 | 27 | 3.34 (1.72-6.50) | <0.001 | 4.10 (1.89-8.89) | <0.001 |
|  |  |  | Yes | High | 10 | 5 | 4.83 (1.29-18.04) | 0.019 | 6.31 (1.40-28.46) | 0.016 |
|  |  |  | No | High | 10 | 6 | 7.24 (1.89-27.72) | 0.004 | 18.15 (3.67-89.83) | <0.001 |
| 7 | Multiple Modes of Instruction Delivery | Regular | Yes | Non-high | 392 | 64 | 1.00 |  | 1.00 |  |
|  |  |  | No | Non-high | 254 | 86 | 2.62 (1.81-3.81) | <0.001 | 2.51 (1.68-3.74) | <0.001 |
|  |  |  | Yes | High | 32 | 12 | 3.08 (1.43-6.60) | 0.004 | 4.06 (1.79-9.22) | 0.001 |
|  |  |  | No | High | 39 | 23 | 7.37 (3.69-14.72) | <0.001 | 8.41 (3.86-18.32) | <0.001 |
|  |  | Non-regular | Yes | Non-high | 112 | 24 | 1.00 |  | 1.00 |  |
|  |  |  | No | Non-high | 88 | 26 | 1.54 (0.81-2.93) | 0.190 | 1.49 (0.72-3.09) | 0.281 |
|  |  |  | Yes | High | 8 | 3 | 2.20 (0.49-9.87) | 0.303 | 3.92 (0.74-20.87) | 0.109 |
|  |  |  | No | High | 12 | 8 | 7.33 (2.03-26.44) | 0.002 | 12.91 (2.82-59.03) | 0.001 |
| 8 | Advance Notice of Schedule Changes | Regular | Yes | Non-high | 337 | 49 | 1.00 |  | 1.00 |  |
|  |  |  | No | Non-high | 309 | 101 | 2.85 (1.94-4.19) | <0.001 | 2.89 (1.91-4.35) | <0.001 |
|  |  |  | Yes | High | 26 | 11 | 4.31 (1.87-9.93) | 0.001 | 5.45 (2.18-13.67) | <0.001 |
|  |  |  | No | High | 45 | 24 | 6.72 (3.47-12.99) | <0.001 | 8.45 (4.04-17.66) | <0.001 |
|  |  | Non-regular | Yes | Non-high | 126 | 22 | 1.00 |  | 1.00 |  |
|  |  |  | No | Non-high | 74 | 28 | 2.88 (1.49-5.55) | 0.002 | 2.74 (1.30-5.80) | 0.008 |
|  |  |  | Yes | High | 6 | 3 | 4.73 (0.89-24.99) | 0.067 | 6.96 (1.07-45.35) | 0.043 |
|  |  |  | No | High | 14 | 8 | 6.30 (1.99-19.99) | 0.002 | 11.21 (2.82-44.52) | 0.001 |
| 9 | Consideration for Task Focus | Regular | Yes | Non-high | 299 | 49 | 1.00 |  | 1.00 |  |
|  |  |  | No | Non-high | 347 | 101 | 2.10 (1.43-3.08) | <0.001 | 2.22 (1.47-3.36) | <0.001 |
|  |  |  | Yes | High | 24 | 7 | 2.10 (0.83-5.34) | 0.118 | 2.95 (1.07-8.12) | 0.037 |
|  |  |  | No | High | 47 | 28 | 7.52 (3.89-14.52) | <0.001 | 9.17 (4.43-19.02) | <0.001 |
|  |  | Non-regular | Yes | Non-high | 121 | 25 | 1.00 |  | 1.00 |  |
|  |  |  | No | Non-high | 79 | 25 | 1.78 (0.93-3.40) | 0.081 | 1.64 (0.79-3.40) | 0.183 |
|  |  |  | Yes | High | 8 | 4 | 3.84 (0.90-16.44) | 0.070 | 4.74 (0.88-25.68) | 0.071 |
|  |  |  | No | High | 12 | 7 | 5.38 (1.57-18.38) | 0.007 | 11.12 (2.61-47.46) | 0.001 |
| 10 | Regular Meetings with Supervisors | Regular | Yes | Non-high | 363 | 56 | 1.00 |  | 1.00 |  |
|  |  |  | No | Non-high | 283 | 94 | 2.73 (1.87-3.98) | <0.001 | 2.38 (1.59-3.56) | <0.001 |
|  |  |  | Yes | High | 34 | 11 | 2.62 (1.21-5.68) | 0.015 | 3.05 (1.33-6.98) | 0.008 |
|  |  |  | No | High | 37 | 24 | 10.12 (4.87-21.06) | <0.001 | 12.41 (5.43-28.35) | <0.001 |
|  |  | Non-regular | Yes | Non-high | 92 | 12 | 1.00 |  | 1.00 |  |
|  |  |  | No | Non-high | 108 | 38 | 3.62 (1.76-7.47) | <0.001 | 5.02 (2.21-11.38) | <0.001 |
|  |  |  | Yes | High | 7 | 3 | 5.00 (0.99-25.15) | 0.051 | 9.50 (1.55-58.34) | 0.015 |
|  |  |  | No | High | 13 | 8 | 10.67 (2.99-38.04) | <0.001 | 29.75 (5.99-147.86) | <0.001 |
| 11 | Task Assignment Considering Individual Strengths | Regular | Yes | Non-high | 340 | 45 | 1.00 |  | 1.00 |  |
|  |  |  | No | Non-high | 306 | 105 | 3.43 (2.31-5.07) | <0.001 | 3.48 (2.29-5.29) | <0.001 |
|  |  |  | Yes | High | 22 | 7 | 3.06 (1.18-7.91) | 0.021 | 4.16 (1.46-11.80) | 0.007 |
|  |  |  | No | High | 49 | 28 | 8.74 (4.58-16.69) | <0.001 | 10.56 (5.15-21.63) | <0.001 |
|  |  | Non-regular | Yes | Non-high | 112 | 21 | 1.00 |  | 1.00 |  |
|  |  |  | No | Non-high | 88 | 29 | 2.13 (1.11-4.08) | 0.023 | 2.05 (1.00-4.20) | 0.051 |
|  |  |  | Yes | High | 5 | 2 | 2.89 (0.45-18.39) | 0.261 | 4.27 (0.48-38.04) | 0.193 |
|  |  |  | No | High | 15 | 9 | 6.50 (2.09-20.26) | 0.001 | 10.82 (2.94-39.79) | <0.001 |
| 12 | Consideration for Sensory Stimuli | Regular | Yes | Non-high | 345 | 49 | 1.00 |  | 1.00 |  |
|  |  |  | No | Non-high | 301 | 101 | 3.05 (2.08-4.49) | <0.001 | 3.02 (2.00-4.58) | <0.001 |
|  |  |  | Yes | High | 25 | 9 | 3.40 (1.42-8.12) | 0.006 | 3.58 (1.38-9.30) | 0.009 |
|  |  |  | No | High | 46 | 26 | 7.85 (4.07-15.14) | <0.001 | 10.31 (4.96-21.46) | <0.001 |
|  |  | Non-regular | Yes | Non-high | 111 | 21 | 1.00 |  | 1.00 |  |
|  |  |  | No | Non-high | 89 | 29 | 2.07 (1.08-3.97) | 0.028 | 2.40 (1.18-4.88) | 0.016 |
|  |  |  | Yes | High | 5 | 2 | 2.86 (0.45-18.19) | 0.266 | 4.55 (0.57-36.36) | 0.154 |
|  |  |  | No | High | 15 | 9 | 6.43 (2.06-20.04) | 0.001 | 13.21 (3.36-51.88) | <0.001 |
| 13 | Active Workplace Communication | Regular | Yes | Non-high | 408 | 62 | 1.00 |  | 1.00 |  |
|  |  |  | No | Non-high | 238 | 88 | 3.27 (2.25-4.77) | <0.001 | 3.43 (2.28-5.16) | <0.001 |
|  |  |  | Yes | High | 32 | 10 | 2.54 (1.15-5.62) | 0.022 | 3.10 (1.31-7.35) | 0.010 |
|  |  |  | No | High | 39 | 25 | 9.97 (4.91-20.23) | <0.001 | 13.69 (6.13-30.55) | <0.001 |
|  |  | Non-regular | Yes | Non-high | 139 | 25 | 1.00 |  | 1.00 |  |
|  |  |  | No | Non-high | 61 | 25 | 3.17 (1.62-6.18) | 0.001 | 4.06 (1.85-8.93) | <0.001 |
|  |  |  | Yes | High | 10 | 3 | 1.95 (0.47-8.09) | 0.355 | 3.29 (0.64-16.8) | 0.153 |
|  |  |  | No | High | 10 | 8 | 18.24 (3.65-91.14) | <0.001 | 54.56 (7.79-382.34) | <0.001 |
| 14 | Training on Diversity and Disability Awareness | Regular | Yes | Non-high | 304 | 51 | 1.00 |  | 1.00 |  |
|  |  |  | No | Non-high | 342 | 99 | 2.02 (1.38-2.96) | <0.001 | 1.81 (1.21-2.71) | 0.004 |
|  |  |  | Yes | High | 29 | 10 | 2.61 (1.15-5.94) | 0.022 | 3.78 (1.53-9.34) | 0.004 |
|  |  |  | No | High | 42 | 25 | 7.30 (3.68-14.48) | <0.001 | 7.09 (3.38-14.89) | <0.001 |
|  |  | Non-regular | Yes | Non-high | 66 | 11 | 1.00 |  | 1.00 |  |
|  |  |  | No | Non-high | 134 | 39 | 2.05 (0.97-4.33) | 0.059 | 1.98 (0.87-4.52) | 0.105 |
|  |  |  | Yes | High | 3 | 1 | 2.50 (0.21-30.04) | 0.470 | 5.16 (0.27-97.83) | 0.275 |
|  |  |  | No | High | 17 | 10 | 7.14 (2.23-22.85) | 0.001 | 10.83 (2.91-40.28) | <0.001 |

Sex (A) ASD traits

| No. | Workplace adjustments | | | ASD  traits | No. of subjects | No. of cases | Univariate | | Multivariable | |  |
| --- | --- | --- | --- | --- | --- | --- | --- | --- | --- | --- | --- |
|  | Type | Sex | Presence |  |  |  | OR (95% CI) | p-value | OR (95% CI) | p-value | |
| 1 | Adjustment of Break Times | Male | Yes | Non-high | 283 | 46 | 1.00 |  | 1.00 |  | |
|  |  |  | No | Non-high | 172 | 60 | 2.76 (1.77-4.31) | <0.001 | 2.82 (1.73-4.59) | <0.001 | |
|  |  |  | Yes | High | 22 | 14 | 9.02 (3.58-22.72) | <0.001 | 8.22 (2.9-23.29) | <0.001 | |
|  |  |  | No | High | 23 | 17 | 14.60 (5.46-39.01) | <0.001 | 12.96 (4.48-37.55) | <0.001 | |
|  |  | Female | Yes | Non-high | 261 | 42 | 1.00 |  | 1.00 |  | |
|  |  |  | No | Non-high | 206 | 72 | 2.80 (1.81-4.34) | <0.001 | 3.18 (1.97-5.13) | <0.001 | |
|  |  |  | Yes | High | 16 | 8 | 5.21 (1.85-14.67) | 0.002 | 6.45 (2.05-20.29) | 0.001 | |
|  |  |  | No | High | 17 | 13 | 16.95 (5.27-54.51) | <0.001 | 17.74 (5.25-59.89) | <0.001 | |
| 2 | Quiet Rest Area | Male | Yes | Non-high | 234 | 41 | 1.00 |  | 1.00 |  | |
|  |  |  | No | Non-high | 221 | 65 | 1.96 (1.26-3.06) | 0.003 | 2.13 (1.32-3.45) | 0.002 | |
|  |  |  | Yes | High | 15 | 8 | 5.38 (1.85-15.67) | 0.002 | 3.98 (1.21-13.16) | 0.023 | |
|  |  |  | No | High | 30 | 23 | 15.47 (6.22-38.45) | <0.001 | 14.94 (5.62-39.69) | <0.001 | |
|  |  | Female | Yes | Non-high | 249 | 41 | 1.00 |  | 1.00 |  | |
|  |  |  | No | Non-high | 218 | 73 | 2.55 (1.65-3.96) | <0.001 | 2.68 (1.67-4.31) | <0.001 | |
|  |  |  | Yes | High | 17 | 9 | 5.71 (2.08-15.66) | 0.001 | 6.85 (2.25-20.85) | 0.001 | |
|  |  |  | No | High | 16 | 12 | 15.22 (4.68-49.54) | <0.001 | 14.97 (4.38-51.22) | <0.001 | |
| 3 | Consideration for Health / Flexible Work Hours | Male | Yes | Non-high | 293 | 51 | 1.00 |  | 1.00 |  | |
|  |  |  | No | Non-high | 162 | 55 | 2.44 (1.57-3.80) | <0.001 | 2.76 (1.68-4.53) | <0.001 | |
|  |  |  | Yes | High | 30 | 18 | 7.12 (3.23-15.69) | <0.001 | 6.31 (2.60-15.30) | <0.001 | |
|  |  |  | No | High | 15 | 13 | 30.84 (6.75-140.88) | <0.001 | 33.24 (6.77-163.27) | <0.001 | |
|  |  | Female | Yes | Non-high | 337 | 62 | 1.00 |  | 1.00 |  | |
|  |  |  | No | Non-high | 130 | 52 | 2.96 (1.89-4.62) | <0.001 | 2.89 (1.80-4.63) | <0.001 | |
|  |  |  | Yes | High | 22 | 12 | 5.32 (2.20-12.87) | <0.001 | 6.20 (2.38-16.18) | <0.001 | |
|  |  |  | No | High | 11 | 9 | 19.96 (4.21-94.68) | <0.001 | 16.45 (3.30-81.86) | 0.001 | |
| 4 | Workload Sharing / Reducing Burden | Male | Yes | Non-high | 267 | 39 | 1.00 |  | 1.00 |  | |
|  |  |  | No | Non-high | 188 | 67 | 3.24 (2.06-5.09) | <0.001 | 3.27 (2.00-5.35) | <0.001 | |
|  |  |  | Yes | High | 22 | 12 | 7.02 (2.84-17.35) | <0.001 | 6.27 (2.29-17.18) | <0.001 | |
|  |  |  | No | High | 23 | 19 | 27.77 (8.97-86.00) | <0.001 | 26.94 (8.07-89.97) | <0.001 | |
|  |  | Female | Yes | Non-high | 261 | 42 | 1.00 |  | 1.00 |  | |
|  |  |  | No | Non-high | 206 | 72 | 2.80 (1.81-4.34) | <0.001 | 2.86 (1.79-4.58) | <0.001 | |
|  |  |  | Yes | High | 17 | 9 | 5.87 (2.14-16.07) | 0.001 | 6.65 (2.20-20.11) | 0.001 | |
|  |  |  | No | High | 16 | 12 | 15.64 (4.81-50.85) | <0.001 | 16.54 (4.80-56.98) | <0.001 | |
| 5 | Dedicated Space for Focused Work | Male | Yes | Non-high | 190 | 31 | 1.00 |  | 1.00 |  | |
|  |  |  | No | Non-high | 265 | 75 | 2.03 (1.27-3.24) | 0.003 | 1.91 (1.14-3.17) | 0.013 | |
|  |  |  | Yes | High | 14 | 8 | 6.84 (2.22-21.09) | 0.001 | 5.37 (1.55-18.61) | 0.008 | |
|  |  |  | No | High | 31 | 23 | 14.75 (6.05-35.97) | <0.001 | 13.04 (4.91-34.63) | <0.001 | |
|  |  | Female | Yes | Non-high | 180 | 25 | 1.00 |  | 1.00 |  | |
|  |  |  | No | Non-high | 287 | 89 | 2.79 (1.71-4.55) | <0.001 | 3.16 (1.85-5.41) | <0.001 | |
|  |  |  | Yes | High | 14 | 7 | 6.20 (2.00-19.19) | 0.002 | 6.23 (1.77-21.91) | 0.004 | |
|  |  |  | No | High | 19 | 14 | 17.36 (5.75-52.42) | <0.001 | 21.50 (6.67-69.34) | <0.001 | |
| 6 | Clear and Specific Work Instructions | Male | Yes | Non-high | 271 | 41 | 1.00 |  | 1.00 |  | |
|  |  |  | No | Non-high | 184 | 65 | 3.06 (1.96-4.80) | <0.001 | 3.62 (2.19-6.00) | <0.001 | |
|  |  |  | Yes | High | 25 | 16 | 9.97 (4.13-24.08) | <0.001 | 8.93 (3.26-24.45) | <0.001 | |
|  |  |  | No | High | 20 | 15 | 16.83 (5.80-48.83) | <0.001 | 18.13 (5.74-57.31) | <0.001 | |
|  |  | Female | Yes | Non-high | 285 | 43 | 1.00 |  | 1.00 |  | |
|  |  |  | No | Non-high | 182 | 71 | 3.60 (2.32-5.59) | <0.001 | 4.62 (2.82-7.56) | <0.001 | |
|  |  |  | Yes | High | 23 | 12 | 6.14 (2.55-14.80) | <0.001 | 7.37 (2.77-19.63) | <0.001 | |
|  |  |  | No | High | 10 | 9 | 50.65 (6.26-410.04) | <0.001 | 58.37 (6.44-529.24) | <0.001 | |
| 7 | Multiple Modes of Instruction Delivery | Male | Yes | Non-high | 248 | 40 | 1.00 |  | 1.00 |  | |
|  |  |  | No | Non-high | 207 | 66 | 2.43 (1.56-3.81) | <0.001 | 2.01 (1.23-3.28) | 0.005 | |
|  |  |  | Yes | High | 20 | 13 | 9.66 (3.63-25.71) | <0.001 | 8.42 (2.86-24.77) | <0.001 | |
|  |  |  | No | High | 25 | 18 | 13.37 (5.24-34.11) | <0.001 | 10.04 (3.64-27.65) | <0.001 | |
|  |  | Female | Yes | Non-high | 285 | 48 | 1.00 |  | 1.00 |  | |
|  |  |  | No | Non-high | 182 | 66 | 2.81 (1.82-4.33) | <0.001 | 2.95 (1.86-4.70) | <0.001 | |
|  |  |  | Yes | High | 18 | 10 | 6.17 (2.32-16.45) | <0.001 | 7.09 (2.42-20.80) | <0.001 | |
|  |  |  | No | High | 15 | 11 | 13.58 (4.15-44.44) | <0.001 | 14.02 (4.00-49.15) | <0.001 | |
| 8 | Advance Notice of Schedule Changes | Male | Yes | Non-high | 234 | 38 | 1.00 |  | 1.00 |  | |
|  |  |  | No | Non-high | 221 | 68 | 2.29 (1.46-3.59) | <0.001 | 2.34 (1.43-3.84) | 0.001 | |
|  |  |  | Yes | High | 17 | 8 | 4.59 (1.66-12.64) | 0.003 | 3.79 (1.22-11.83) | 0.022 | |
|  |  |  | No | High | 28 | 23 | 23.73 (8.49-66.30) | <0.001 | 21.60 (7.22-64.67) | <0.001 | |
|  |  | Female | Yes | Non-high | 260 | 39 | 1.00 |  | 1.00 |  | |
|  |  |  | No | Non-high | 207 | 75 | 3.22 (2.07-5.01) | <0.001 | 3.37 (2.08-5.43) | <0.001 | |
|  |  |  | Yes | High | 19 | 10 | 6.30 (2.40-16.49) | <0.001 | 6.29 (2.21-17.92) | 0.001 | |
|  |  |  | No | High | 14 | 11 | 20.78 (5.54-77.87) | <0.001 | 24.52 (6.03-99.67) | <0.001 | |
| 9 | Consideration for Task Focus | Male | Yes | Non-high | 220 | 34 | 1.00 |  | 1.00 |  | |
|  |  |  | No | Non-high | 235 | 72 | 2.42 (1.53-3.82) | <0.001 | 2.56 (1.54-4.24) | <0.001 | |
|  |  |  | Yes | High | 20 | 11 | 6.69 (2.58-17.35) | <0.001 | 4.99 (1.71-14.56) | 0.003 | |
|  |  |  | No | High | 25 | 20 | 21.88 (7.69-62.28) | <0.001 | 22.81 (7.44-69.94) | <0.001 | |
|  |  | Female | Yes | Non-high | 237 | 44 | 1.00 |  | 1.00 |  | |
|  |  |  | No | Non-high | 230 | 70 | 1.92 (1.25-2.95) | 0.003 | 2.13 (1.33-3.42) | 0.002 | |
|  |  |  | Yes | High | 14 | 7 | 4.39 (1.46-13.15) | 0.008 | 4.34 (1.30-14.48) | 0.017 | |
|  |  |  | No | High | 19 | 14 | 12.28 (4.20-35.89) | <0.001 | 15.11 (4.79-47.67) | <0.001 | |
| 10 | Regular Meetings with Supervisors | Male | Yes | Non-high | 241 | 33 | 1.00 |  | 1.00 |  | |
|  |  |  | No | Non-high | 214 | 73 | 3.26 (2.05-5.19) | <0.001 | 2.88 (1.75-4.74) | <0.001 | |
|  |  |  | Yes | High | 21 | 11 | 6.93 (2.73-17.6) | <0.001 | 6.55 (2.38-18.03) | <0.001 | |
|  |  |  | No | High | 24 | 20 | 31.52 (10.13-98.01) | <0.001 | 22.66 (6.69-76.79) | <0.001 | |
|  |  | Female | Yes | Non-high | 238 | 33 | 1.00 |  | 1.00 |  | |
|  |  |  | No | Non-high | 229 | 81 | 3.4 (2.15-5.37) | <0.001 | 3.62 (2.22-5.89) | <0.001 | |
|  |  |  | Yes | High | 18 | 10 | 7.77 (2.86-21.1) | <0.001 | 8.84 (2.96-26.4) | <0.001 | |
|  |  |  | No | High | 15 | 11 | 17.08 (5.14-56.83) | <0.001 | 20.2 (5.57-73.31) | <0.001 | |
| 11 | Task Assignment Considering Individual Strengths | Male | Yes | Non-high | 232 | 35 | 1.00 |  | 1.00 |  | |
|  |  |  | No | Non-high | 223 | 71 | 2.63 (1.67-4.15) | <0.001 | 2.85 (1.73-4.71) | <0.001 | |
|  |  |  | Yes | High | 13 | 5 | 3.52 (1.09-11.38) | 0.036 | 2.96 (0.79-11.02) | 0.106 | |
|  |  |  | No | High | 32 | 26 | 24.39 (9.36-63.56) | <0.001 | 22.48 (8.03-62.99) | <0.001 | |
|  |  | Female | Yes | Non-high | 256 | 39 | 1.00 |  | 1.00 |  | |
|  |  |  | No | Non-high | 211 | 75 | 3.07 (1.97-4.78) | <0.001 | 3.08 (1.92-4.93) | <0.001 | |
|  |  |  | Yes | High | 14 | 7 | 5.56 (1.85-16.74) | 0.002 | 6.57 (1.97-21.95) | 0.002 | |
|  |  |  | No | High | 19 | 14 | 15.58 (5.31-45.72) | <0.001 | 15.54 (4.98-48.53) | <0.001 | |
| 12 | Consideration for Sensory Stimuli | Male | Yes | Non-high | 240 | 38 | 1.00 |  | 1.00 |  | |
|  |  |  | No | Non-high | 215 | 68 | 2.46 (1.57-3.86) | <0.001 | 2.45 (1.49-4.01) | <0.001 | |
|  |  |  | Yes | High | 17 | 10 | 7.59 (2.72-21.19) | <0.001 | 8.27 (2.59-26.46) | <0.001 | |
|  |  |  | No | High | 28 | 21 | 15.95 (6.34-40.13) | <0.001 | 12.30 (4.59-33.00) | <0.001 | |
|  |  | Female | Yes | Non-high | 250 | 36 | 1.00 |  | 1.00 |  | |
|  |  |  | No | Non-high | 217 | 78 | 3.34 (2.13-5.23) | <0.001 | 3.70 (2.28-5.99) | <0.001 | |
|  |  |  | Yes | High | 16 | 8 | 5.94 (2.10-16.85) | 0.001 | 6.04 (1.93-18.88) | 0.002 | |
|  |  |  | No | High | 17 | 13 | 19.32 (5.97-62.56) | <0.001 | 24.7 (7.05-86.51) | <0.001 | |
| 13 | Active Workplace Communication | Male | Yes | Non-high | 273 | 43 | 1.00 |  | 1.00 |  | |
|  |  |  | No | Non-high | 182 | 63 | 2.83 (1.81-4.43) | <0.001 | 2.82 (1.73-4.61) | <0.001 | |
|  |  |  | Yes | High | 21 | 11 | 5.88 (2.35-14.71) | <0.001 | 4.91 (1.77-13.61) | 0.002 | |
|  |  |  | No | High | 24 | 20 | 26.74 (8.71-82.12) | <0.001 | 25.32 (7.59-84.43) | <0.001 | |
|  |  | Female | Yes | Non-high | 311 | 44 | 1.00 |  | 1.00 |  | |
|  |  |  | No | Non-high | 156 | 70 | 4.94 (3.15-7.74) | <0.001 | 5.40 (3.31-8.81) | <0.001 | |
|  |  |  | Yes | High | 18 | 10 | 7.59 (2.84-20.27) | <0.001 | 8.15 (2.71-24.47) | <0.001 | |
|  |  |  | No | High | 15 | 11 | 16.69 (5.09-54.74) | <0.001 | 18.11 (5.14-63.83) | <0.001 | |
| 14 | Training on Diversity and Disability Awareness | Male | Yes | Non-high | 199 | 32 | 1.00 |  | 1.00 |  | |
|  |  |  | No | Non-high | 256 | 74 | 2.12 (1.33-3.38) | 0.002 | 1.55 (0.94-2.57) | 0.088 | |
|  |  |  | Yes | High | 15 | 8 | 5.96 (2.02-17.61) | 0.001 | 4.83 (1.49-15.66) | 0.009 | |
|  |  |  | No | High | 30 | 23 | 17.15 (6.79-43.32) | <0.001 | 11.33 (4.16-30.85) | <0.001 | |
|  |  | Female | Yes | Non-high | 195 | 33 | 1.00 |  | 1.00 |  | |
|  |  |  | No | Non-high | 272 | 81 | 2.08 (1.32-3.28) | 0.002 | 2.14 (1.31-3.51) | 0.003 | |
|  |  |  | Yes | High | 10 | 4 | 3.27 (0.88-12.24) | 0.078 | 3.72 (0.90-15.44) | 0.070 | |
|  |  |  | No | High | 23 | 17 | 13.91 (5.10-37.93) | <0.001 | 14.97 (5.11-43.81) | <0.001 | |

Sex (B) ADHD traits

| No. | Workplace adjustments | | | | ADHD | No. of subjects | No. of cases | Univariate | | | Multivariable | | |
| --- | --- | --- | --- | --- | --- | --- | --- | --- | --- | --- | --- | --- | --- |
|  | Type | Sex | Presence | traits | |  |  | OR (95% CI) | p-value | OR (95% CI) | | p-value |  |
| 1 | Adjustment of Break Times | Male | Yes | Non-high | | 285 | 52 | 1.00 |  | 1.00 | |  |  |
|  |  |  | No | Non-high | | 164 | 55 | 2.26 (1.45-3.52) | <0.001 | 2.25 (1.38-3.66) | | 0.001 |  |
|  |  |  | Yes | High | | 20 | 8 | 2.99 (1.16-7.68) | 0.023 | 2.96 (1.03-8.49) | | 0.044 |  |
|  |  |  | No | High | | 31 | 22 | 10.95 (4.77-25.16) | <0.001 | 10.15 (4.09-25.20) | | <0.001 |  |
|  |  | Female | Yes | Non-high | | 255 | 42 | 1.00 |  | 1.00 | |  |  |
|  |  |  | No | Non-high | | 198 | 72 | 2.90 (1.87-4.50) | <0.001 | 3.28 (2.03-5.30) | | <0.001 |  |
|  |  |  | Yes | High | | 22 | 8 | 2.90 (1.14-7.34) | 0.025 | 4.76 (1.66-13.59) | | 0.004 |  |
|  |  |  | No | High | | 25 | 13 | 5.49 (2.35-12.87) | <0.001 | 8.07 (3.14-20.71) | | <0.001 |  |
| 2 | Quiet Rest Area | Male | Yes | Non-high | | 233 | 44 | 1.00 |  | 1.00 | |  |  |
|  |  |  | No | Non-high | | 216 | 63 | 1.77 (1.14-2.75) | 0.011 | 1.89 (1.17-3.05) | | 0.009 |  |
|  |  |  | Yes | High | | 16 | 5 | 1.95 (0.65-5.91) | 0.236 | 1.53 (0.44-5.31) | | 0.501 |  |
|  |  |  | No | High | | 35 | 25 | 10.74 (4.81-23.98) | <0.001 | 11.36 (4.70-27.48) | | <0.001 |  |
|  |  | Female | Yes | Non-high | | 243 | 44 | 1.00 |  | 1.00 | |  |  |
|  |  |  | No | Non-high | | 210 | 70 | 2.26 (1.46-3.49) | <0.001 | 2.40 (1.50-3.83) | | <0.001 |  |
|  |  |  | Yes | High | | 23 | 6 | 1.60 (0.60-4.28) | 0.353 | 2.48 (0.85-7.25) | | 0.098 |  |
|  |  |  | No | High | | 24 | 15 | 7.54 (3.10-18.33) | <0.001 | 11.26 (4.12-30.8) | | <0.001 |  |
| 3 | Consideration for Health / Flexible Work Hours | Male | Yes | Non-high | | 295 | 56 | 1.00 |  | 1.00 | |  |  |
|  |  |  | No | Non-high | | 154 | 51 | 2.11 (1.36-3.30) | 0.001 | 2.33 (1.42-3.84) | | 0.001 |  |
|  |  |  | Yes | High | | 28 | 13 | 3.70 (1.67-8.21) | 0.001 | 3.41 (1.43-8.13) | | 0.006 |  |
|  |  |  | No | High | | 23 | 17 | 12.09 (4.56-32.06) | <0.001 | 16.12 (5.13-50.61) | | <0.001 |  |
|  |  | Female | Yes | Non-high | | 331 | 65 | 1.00 |  | 1.00 | |  |  |
|  |  |  | No | Non-high | | 122 | 49 | 2.75 (1.75-4.32) | <0.001 | 2.71 (1.67-4.38) | | <0.001 |  |
|  |  |  | Yes | High | | 28 | 9 | 1.94 (0.84-4.48) | 0.122 | 3.21 (1.25-8.25) | | 0.015 |  |
|  |  |  | No | High | | 19 | 12 | 7.02 (2.66-18.52) | <0.001 | 8.13 (2.82-23.46) | | <0.001 |  |
| 4 | Workload Sharing / Reducing Burden | Male | Yes | Non-high | | 270 | 42 | 1.00 |  | 1.00 | |  |  |
|  |  |  | No | Non-high | | 179 | 65 | 3.10 (1.98-4.85) | <0.001 | 3.06 (1.89-4.97) | | <0.001 |  |
|  |  |  | Yes | High | | 19 | 9 | 4.89 (1.87-12.75) | 0.001 | 5.02 (1.73-14.53) | | 0.003 |  |
|  |  |  | No | High | | 32 | 21 | 10.36 (4.66-23.07) | <0.001 | 9.51 (3.95-22.92) | | <0.001 |  |
|  |  | Female | Yes | Non-high | | 262 | 47 | 1.00 |  | 1.00 | |  |  |
|  |  |  | No | Non-high | | 191 | 67 | 2.47 (1.60-3.81) | <0.001 | 2.39 (1.50-3.82) | | <0.001 |  |
|  |  |  | Yes | High | | 16 | 4 | 1.53 (0.47-4.94) | 0.482 | 1.95 (0.53-7.20) | | 0.316 |  |
|  |  |  | No | High | | 31 | 17 | 5.56 (2.56-12.05) | <0.001 | 8.14 (3.47-19.09) | | <0.001 |  |
| 5 | Dedicated Space for Focused Work | Male | Yes | Non-high | | 187 | 31 | 1.00 |  | 1.00 | |  |  |
|  |  |  | No | Non-high | | 262 | 76 | 2.06 (1.29-3.29) | 0.003 | 1.82 (1.10-3.03) | | 0.020 |  |
|  |  |  | Yes | High | | 17 | 8 | 4.47 (1.60-12.50) | 0.004 | 3.73 (1.16-12.00) | | 0.027 |  |
|  |  |  | No | High | | 34 | 22 | 9.23 (4.14-20.57) | <0.001 | 8.25 (3.46-19.67) | | <0.001 |  |
|  |  | Female | Yes | Non-high | | 184 | 30 | 1.00 |  | 1.00 | |  |  |
|  |  |  | No | Non-high | | 269 | 84 | 2.33 (1.46-3.72) | <0.001 | 2.62 (1.57-4.39) | | <0.001 |  |
|  |  |  | Yes | High | | 10 | 2 | 1.28 (0.26-6.34) | 0.760 | 1.46 (0.26-8.26) | | 0.672 |  |
|  |  |  | No | High | | 37 | 19 | 5.42 (2.55-11.52) | <0.001 | 9.39 (3.96-22.27) | | <0.001 |  |
| 6 | Clear and Specific Work Instructions | Male | Yes | Non-high | | 269 | 44 | 1.00 |  | 1.00 | |  |  |
|  |  |  | No | Non-high | | 180 | 63 | 2.75 (1.76-4.30) | <0.001 | 3.09 (1.89-5.06) | | <0.001 |  |
|  |  |  | Yes | High | | 27 | 13 | 4.75 (2.09-10.79) | <0.001 | 4.17 (1.65-10.53) | | 0.003 |  |
|  |  |  | No | High | | 24 | 17 | 12.42 (4.86-31.72) | <0.001 | 14.81 (5.23-41.96) | | <0.001 |  |
|  |  | Female | Yes | Non-high | | 286 | 46 | 1.00 |  | 1.00 | |  |  |
|  |  |  | No | Non-high | | 167 | 68 | 3.58 (2.31-5.57) | <0.001 | 4.40 (2.69-7.21) | | <0.001 |  |
|  |  |  | Yes | High | | 22 | 9 | 3.61 (1.46-8.94) | 0.005 | 4.76 (1.67-13.53) | | 0.003 |  |
|  |  |  | No | High | | 25 | 12 | 4.82 (2.07-11.22) | <0.001 | 9.53 (3.65-24.85) | | <0.001 |  |
| 7 | Multiple Modes of Instruction Delivery | Male | Yes | Non-high | | 250 | 45 | 1.00 |  | 1.00 | |  |  |
|  |  |  | No | Non-high | | 199 | 62 | 2.06 (1.33-3.20) | 0.001 | 1.63 (1.01-2.63) | | 0.047 |  |
|  |  |  | Yes | High | | 18 | 8 | 3.64 (1.36-9.75) | 0.010 | 3.34 (1.13-9.86) | | 0.029 |  |
|  |  |  | No | High | | 33 | 22 | 9.11 (4.13-20.12) | <0.001 | 7.55 (3.13-18.19) | | <0.001 |  |
|  |  | Female | Yes | Non-high | | 280 | 50 | 1.00 |  | 1.00 | |  |  |
|  |  |  | No | Non-high | | 173 | 64 | 2.70 (1.75-4.17) | <0.001 | 2.87 (1.79-4.60) | | <0.001 |  |
|  |  |  | Yes | High | | 23 | 8 | 2.45 (0.99-6.10) | 0.053 | 4.23 (1.52-11.77) | | 0.006 |  |
|  |  |  | No | High | | 24 | 13 | 5.44 (2.30-12.84) | <0.001 | 7.07 (2.74-18.20) | | <0.001 |  |
| 8 | Advance Notice of Schedule Changes | Male | Yes | Non-high | | 235 | 37 | 1.00 |  | 1.00 | |  |  |
|  |  |  | No | Non-high | | 214 | 70 | 2.60 (1.66-4.09) | <0.001 | 2.55 (1.55-4.18) | | <0.001 |  |
|  |  |  | Yes | High | | 16 | 9 | 6.88 (2.41-19.63) | <0.001 | 5.51 (1.72-17.63) | | 0.004 |  |
|  |  |  | No | High | | 35 | 21 | 8.03 (3.75-17.20) | <0.001 | 8.39 (3.59-19.63) | | <0.001 |  |
|  |  | Female | Yes | Non-high | | 260 | 42 | 1.00 |  | 1.00 | |  |  |
|  |  |  | No | Non-high | | 193 | 72 | 3.09 (1.99-4.80) | <0.001 | 3.29 (2.03-5.31) | | <0.001 |  |
|  |  |  | Yes | High | | 19 | 7 | 3.03 (1.13-8.14) | 0.028 | 5.01 (1.64-15.32) | | 0.005 |  |
|  |  |  | No | High | | 28 | 14 | 5.19 (2.31-11.68) | <0.001 | 7.56 (3.04-18.82) | | <0.001 |  |
| 9 | Consideration for Task Focus | Male | Yes | Non-high | | 221 | 38 | 1.00 |  | 1.00 | |  |  |
|  |  |  | No | Non-high | | 228 | 69 | 2.09 (1.33-3.28) | 0.001 | 2.17 (1.32-3.55) | | 0.002 |  |
|  |  |  | Yes | High | | 19 | 7 | 2.81 (1.04-7.60) | 0.042 | 2.24 (0.73-6.89) | | 0.159 |  |
|  |  |  | No | High | | 32 | 23 | 12.31 (5.28-28.68) | <0.001 | 12.99 (5.14-32.8) | | <0.001 |  |
|  |  | Female | Yes | Non-high | | 234 | 45 | 1.00 |  | 1.00 | |  |  |
|  |  |  | No | Non-high | | 219 | 69 | 1.93 (1.25-2.98) | 0.003 | 2.14 (1.33-3.44) | | 0.002 |  |
|  |  |  | Yes | High | | 17 | 6 | 2.29 (0.80-6.52) | 0.121 | 3.13 (0.96-10.20) | | 0.058 |  |
|  |  |  | No | High | | 30 | 15 | 4.20 (1.91-9.22) | <0.001 | 6.77 (2.82-16.24) | | <0.001 |  |
| 10 | Regular Meetings with Supervisors | Male | Yes | Non-high | | 242 | 36 | 1.00 |  | 1.00 | |  |  |
|  |  |  | No | Non-high | | 207 | 71 | 2.99 (1.89-4.71) | <0.001 | 2.54 (1.55-4.15) | | <0.001 |  |
|  |  |  | Yes | High | | 20 | 8 | 3.82 (1.46-9.98) | 0.006 | 3.69 (1.28-10.62) | | 0.015 |  |
|  |  |  | No | High | | 31 | 22 | 13.99 (5.96-32.81) | <0.001 | 11.45 (4.47-29.33) | | <0.001 |  |
|  |  | Female | Yes | Non-high | | 234 | 37 | 1.00 |  | 1.00 | |  |  |
|  |  |  | No | Non-high | | 219 | 77 | 2.89 (1.85-4.52) | <0.001 | 2.96 (1.83-4.78) | | <0.001 |  |
|  |  |  | Yes | High | | 22 | 6 | 2.00 (0.73-5.44) | 0.176 | 2.71 (0.91-8.03) | | 0.072 |  |
|  |  |  | No | High | | 25 | 15 | 7.99 (3.33-19.14) | <0.001 | 12.4 (4.70-32.69) | | <0.001 |  |
| 11 | Task Assignment Considering Individual Strengths | Male | Yes | Non-high | | 231 | 35 | 1.00 |  | 1.00 | |  |  |
|  |  |  | No | Non-high | | 218 | 72 | 2.76 (1.75-4.36) | <0.001 | 2.78 (1.69-4.58) | | <0.001 |  |
|  |  |  | Yes | High | | 14 | 5 | 3.11 (0.98-9.83) | 0.053 | 2.02 (0.53-7.73) | | 0.303 |  |
|  |  |  | No | High | | 37 | 25 | 11.67 (5.37-25.37) | <0.001 | 12.28 (5.19-29.01) | | <0.001 |  |
|  |  | Female | Yes | Non-high | | 253 | 40 | 1.00 |  | 1.00 | |  |  |
|  |  |  | No | Non-high | | 200 | 74 | 3.13 (2.01-4.87) | <0.001 | 2.99 (1.86-4.80) | | <0.001 |  |
|  |  |  | Yes | High | | 17 | 6 | 2.91 (1.02-8.30) | 0.047 | 3.72 (1.13-12.22) | | 0.031 |  |
|  |  |  | No | High | | 30 | 15 | 5.33 (2.41-11.75) | <0.001 | 7.62 (3.20-18.16) | | <0.001 |  |
| 12 | Consideration for Sensory Stimuli | Male | Yes | Non-high | | 238 | 40 | 1.00 |  | 1.00 | |  |  |
|  |  |  | No | Non-high | | 211 | 67 | 2.30 (1.47-3.60) | <0.001 | 2.07 (1.27-3.37) | | 0.004 |  |
|  |  |  | Yes | High | | 19 | 8 | 3.60 (1.36-9.52) | 0.010 | 2.73 (0.93-7.98) | | 0.067 |  |
|  |  |  | No | High | | 32 | 22 | 10.89 (4.79-24.75) | <0.001 | 11.44 (4.52-28.94) | | <0.001 |  |
|  |  | Female | Yes | Non-high | | 252 | 40 | 1.00 |  | 1.00 | |  |  |
|  |  |  | No | Non-high | | 201 | 74 | 3.09 (1.98-4.81) | <0.001 | 3.35 (2.08-5.41) | | <0.001 |  |
|  |  |  | Yes | High | | 14 | 4 | 2.12 (0.63-7.09) | 0.223 | 3.05 (0.80-11.68) | | 0.104 |  |
|  |  |  | No | High | | 33 | 17 | 5.63 (2.63-12.06) | <0.001 | 8.75 (3.71-20.64) | | <0.001 |  |
| 13 | Active Workplace Communication | Male | Yes | Non-high | | 275 | 47 | 1.00 |  | 1.00 | |  |  |
|  |  |  | No | Non-high | | 174 | 60 | 2.55 (1.64-3.98) | <0.001 | 2.50 (1.53-4.07) | | <0.001 |  |
|  |  |  | Yes | High | | 19 | 7 | 2.83 (1.06-7.57) | 0.038 | 2.79 (0.97-8.08) | | 0.058 |  |
|  |  |  | No | High | | 32 | 23 | 12.40 (5.39-28.49) | <0.001 | 11.65 (4.59-29.58) | | <0.001 |  |
|  |  | Female | Yes | Non-high | | 305 | 48 | 1.00 |  | 1.00 | |  |  |
|  |  |  | No | Non-high | | 148 | 66 | 4.31 (2.76-6.74) | <0.001 | 4.76 (2.92-7.78) | | <0.001 |  |
|  |  |  | Yes | High | | 24 | 6 | 1.79 (0.67-4.73) | 0.244 | 2.64 (0.90-7.75) | | 0.077 |  |
|  |  |  | No | High | | 23 | 15 | 10.04 (4.03-24.98) | <0.001 | 15.98 (5.84-43.68) | | <0.001 |  |
| 14 | Training on Diversity and Disability Awareness | Male | Yes | Non-high | | 199 | 36 | 1.00 |  | 1.00 | |  |  |
|  |  |  | No | Non-high | | 250 | 71 | 1.80 (1.14-2.83) | 0.011 | 1.30 (0.79-2.14) | | 0.296 |  |
|  |  |  | Yes | High | | 15 | 4 | 1.65 (0.50-5.47) | 0.415 | 1.58 (0.43-5.73) | | 0.490 |  |
|  |  |  | No | High | | 36 | 26 | 11.77 (5.22-26.56) | <0.001 | 8.60 (3.53-20.94) | | <0.001 |  |
|  |  | Female | Yes | Non-high | | 188 | 30 | 1.00 |  | 1.00 | |  |  |
|  |  |  | No | Non-high | | 265 | 84 | 2.44 (1.53-3.90) | <0.001 | 2.58 (1.55-4.29) | | <0.001 |  |
|  |  |  | Yes | High | | 17 | 7 | 3.69 (1.30-10.45) | 0.014 | 7.99 (2.45-26.08) | | 0.001 |  |
|  |  |  | No | High | | 30 | 14 | 4.61 (2.04-10.43) | <0.001 | 5.57 (2.26-13.73) | | <0.001 |  |

ASD: autism spectrum disorder; ADHD: attention-deficit/hyperactivity disorder; OR: odds ratio; CI: confidence interval; High: AQ ≥ 33 (ASD traits)/ ASRS ≥ 4 (ADHD traits); Non-high: AQ < 33 (ASD traits)/ ASRS < 4 (ADHD traits); No. of cases: Number of individuals with workplace maladjustment; Logistic regression analysis was conducted to estimate the ORs and 95% CIs for workplace maladjustment. The multivariable-adjusted model was adjusted for age, sex, marital status, annual household income, educational attainment, job type, occupational position, smoking status, drinking frequency, and leisure-time physical activity.
